# *Bacillus velezensis* LoaP promotes antitermination by antagonizing NusA

**DOI:** 10.1128/mbio.01429-25

**Published:** 2025-10-28

**Authors:** Madison D. Jermain, Thao Tran, Conor C. Jenkins, Benjamin H. Nasisi, Wade C. Winkler

**Affiliations:** 1 Department of Cell Biology and Molecular Genetics, University of Maryland 1068 , College Park, Maryland, USA; 2 Department of Chemistry and Biochemistry, University of Maryland 1068 , College Park, Maryland, USA; The Pennsylvania State University, University Park, Pennsylvania, USA

**Keywords:** antitermination, RNA, transcription elongation, NusG, RNA-binding proteins, secondary metabolites

## Abstract

The transcription factor NusG is essential for the bacterial transcription elongation complex (TEC). During elongation, NusG dynamically interacts with other proteins, affecting transcription-translation coupling, processivity, and termination. NusG is integral to antitermination complexes, where it associates with other factors to modify the TEC, allowing it to bypass termination sites. In Gammaproteobacteria, these complexes enhance the expression of ribosomal RNA operons, bacteriophage operons, and CRISPR arrays. Specialized NusG paralogs, such as LoaP, RfaH, and UpxY, are believed to form their own unique antitermination complexes. RfaH, the only well-studied paralog, binds to a paused TEC via a specific nontemplate DNA sequence. The RfaH-bound TEC then enhances transcription-translation coupling while also preventing Rho termination. However, it is unclear whether the other NusG paralogs use similar mechanisms, nor have antitermination complexes been investigated for Gram-positive bacteria. Previously, *Bacillus velezensis* LoaP was shown to enhance transcription of the difficidin antibiotic synthesis operon, but its antitermination determinants were unknown. In this study, we purified RNA polymerase and transcription factors and reconstituted difficidin operon (*dfn*) antitermination activity *in vitro*. The *dfn* 5′ leader region contains a small RNA hairpin essential for antitermination *in vivo* and *in vitro*, and an intrinsic terminator that is highly dependent on NusA. The antitermination activity of LoaP emanates from its ability to specifically antagonize NusA; no other factors are required. These findings demonstrate the basic requirements of the LoaP class of NusG paralogs and indicate that the LoaP antitermination mechanism differs significantly from that of the RfaH paradigm.

**IMPORTANCE:**

Genes encoding the NusG paralog LoaP are widely found in Bacillota, Actinomycetota, and Spirochaetota, where they are located near biosynthetic operons for secondary metabolites. Previously, *Bacillus velezensis* LoaP was shown to promote readthrough of transcription termination sites located within operons encoding for the biosynthesis of the antibiotics difficidin and macrolactin. Thus, some secondary metabolites are produced from terminator-laden operons that rely on antitermination factors such as LoaP. In this study, we show that LoaP bypasses termination by specifically antagonizing the transcription factor NusA. This demonstrates that the LoaP antitermination complex is simpler than expected and that additional factors are not required. Together, our data expand the repertoire of NusG specialized paralogs and the antitermination mechanisms they utilize. Understanding this molecular mechanism will aid in the discovery of natural products regulated by LoaP proteins and assist in the development of new synthetic biology tools that improve the heterologous expression of natural products.

## INTRODUCTION

During transcription, each nucleotide incorporation cycle can be viewed as a decision between synthesis, pausing, arrest, and termination. Termination signals are typically located at the ends of operons (1). However, when they occur within gene clusters, they often function as key regulatory checkpoints (2). Transcription termination often occurs when the TEC is forced to dissociate from the DNA template and nascent RNA after encountering the Rho termination factor (1, 3–5), which is found widely in bacterial genomes. In Gammaproteobacteria such as *Escherichia coli*, the gene encoding Rho is essential (3). In contrast, many Bacillota, such as *Bacillus subtilis*, display only minor or moderate phenotypes after deletion of the gene encoding Rho (3, 6). Instead, transcription termination typically occurs for these organisms when TECs encounter intrinsic terminator hairpins (1, 7). These RNA elements comprise a G/C-rich helix followed by a polyuridine tract. The polyuridine sequence causes the TEC to pause, which allows the terminator hairpin to form within the RNA exit tunnel of the RNA polymerase (RNAP), ultimately triggering release of the DNA template and nascent RNA (7). Intrinsic terminators are ubiquitous in the *B. subtilis* genome, typically located within riboswitch leader regions or at the ends of operons (8), and are often enhanced by the universal transcription elongation factor, NusA (9, 10).

The fact that *E. coli* relies heavily on Rho for termination, while *B. subtilis* depends more on intrinsic terminator hairpins, suggests that there may be key differences in their mechanisms of transcription elongation. Both organisms rely on similar RNA polymerase proteins, including elongation factors NusA and NusG, yet there are striking differences in the roles of these elongation factors. NusG, which is universally found in all bacteria, has an N-terminal domain (NTD) that associates with the TEC near the upstream DNA helix (11, 12). The *Ec*NusG protein reduces pausing of the *E. coli* TEC, thereby increasing the overall transcription rate (13). In contrast, *Bs*NusG enhances many pause sites across the *B. subtilis* genome in a sequence-specific manner (14–16). The *Ec*NusG C-terminal domain (CTD) extends from the polymerase surface (17–19) to act as a protein-protein interaction hub, associating in a mutually exclusive manner with either ribosomal protein S10 or Rho (18–21). Structural data suggest the protein-protein complex formed by *Ec*NusG and *Ec*S10 acts as a bridge between the TEC and a leading ribosome (22–24) , helping to couple transcription with translation (25). The idea that the TEC is transiently or fully linked to a leading ribosome provides a compelling model in which the close proximity of the leading ribosome to the TEC discourages formation of terminator hairpins. This model also suggests that the NusG-S10 interaction prevents Rho from accessing the *Ec*NusG-CTD, thereby reducing the overall degree of Rho termination. This differs from *B. subtilis*, which appears to employ Rho mostly as a means of preventing antisense transcription and which routinely places intrinsic terminators near stop codons (26). Indeed, the global *in vivo* rates of *E. coli* transcription and translation match well, whereas in *B. subtilis,* transcription rates are double that of the translation rate (26). Based in part on these data, it has been argued that transcription and translation are uncoupled in *B. subtilis*, in striking contrast to that in *E. coli*. This implies that these organisms are likely to use fundamentally different mechanisms for the genetic regulation of transcription elongation.

The *E. coli* TEC can be modified into an antitermination complex that is resistant to Rho, even as the polymerase traverses unusually long DNA sequences (27). Unlike transcription attenuation mechanisms, such as riboswitches, where individual termination regions are bypassed in response to selective cellular conditions (28), modification of the TEC to a terminator-resistant form is sometimes referred to as processive antitermination (27, 29), although it will herein be referred to as “antitermination.” Several classes of *E. coli* antitermination complexes have been investigated. For example, lambdoid phages utilize multiple antitermination mechanisms to enhance the gene expression of transcripts produced during the life cycle of bacteriophage infection (30). For early gene expression, phage λN modifies the TEC such that it blocks Rho access and prevents the dissociation of RNAP during synthesis of the 20-kilobase transcript. This antitermination complex also requires the participation of NusG, NusA, S10, and NusB, an elongation factor that associates with S10. These elongation factors are recruited to the TEC by a few RNA determinants within the nascent mRNA, such as *boxA*, which associates with S10 and NusB, and *boxB*, which recruits λN (31–33). Structural analyses revealed that these ribonucleoprotein complexes shift the placement of the termination factor NusA, so that it has reduced access to the RNA exit tunnel. They also prevent Rho from accessing NusG (31, 32). A similar mechanism is responsible for antitermination of *E. coli* ribosomal RNA (*rrn*) operons. While the *rrn* antitermination complex does not associate with λN, it requires the presence of *boxA*, NusB, S10, NusA, NusG, and an inositol monophosphatase called SuhB (34–37). Like the λN complex, the *rrn* antitermination complex forms a ribonucleoprotein structure that remains associated with the TEC during synthesis of the *rrn* operon and that blocks access by Rho (38). In addition, since CRISPR RNA arrays consist of long noncoding sequences that might otherwise be deleteriously affected by Rho, their transcription is also protected by the *rrn* antitermination complex (39).

Although all bacteria encode a core NusG, many bacteria also encode one or more specialized NusG paralogs (40, 41). These genes can be assigned to several paralogous families, such as TaA, which is primarily encoded by myxobacteria (42–44); UpxY, which is encoded by Bacteroidetes (45, 46); RfaH, encoded by proteobacteria (40, 47, 48); ActX, which is primarily located on conjugative plasmids (49–52); and LoaP, which is found in Bacillota, Actinomycetota, and Spirochaetota (53–55). These specialized NusG paralogs are assumed to act on the TEC as it transcribes specific operons within a targeted regulon. Therefore, each of these NusG paralogs is likely to direct the formation of its own unique antitermination complexes. Among the NusG-specialized paralogs that have been discovered, RfaH has been investigated in the greatest molecular detail. *E. coli* RfaH is recruited to a paused TEC complex by binding to a specific DNA sequence (“*ops”*), displayed in the nontemplate DNA strand of the transcription bubble (56, 57). Once bound, *Ec*RfaH interacts with the TEC through its NTD, similar to *Ec*NusG (11). The *Ec*RfaH-CTD is thought to improve coupling between the TEC and leading ribosome, enhancing translation of the targeted genes, which generally feature poor ribosome-binding sites and alternative start codons (58, 59). Crucially, the *Ec*RfaH-CTD is incapable of binding to Rho (60), thus conferring antitermination of RfaH-targeted operons, including genes for outer membrane functions and lipopolysaccharide biosynthesis.

The antitermination complexes governed by NusG specialized paralogs are undoubtedly different from one bacterium to another, both in their structural and mechanistic requirements. However, the LoaP mechanism must particularly differ from that of RfaH, given that it is primarily found in Gram-positive bacteria (54), which rely more on intrinsic terminator hairpins than Rho termination. While the *E. coli* antitermination complexes act primarily to block access to Rho and, in the case of RfaH, improve transcription-translation coupling, *Bacillus* species neither exhibit a high reliance on Rho nor couple transcription with translation (3, 26); therefore, the LoaP mechanism should differ entirely from all known antitermination complexes.

In this study, we employed proteomic and metabolomic analyses to investigate the *Bacillus velezensis* LoaP regulon. This provided conclusive evidence that LoaP selectively regulates the production of polyketide synthase enzymes for the antibiotics difficidin and macrolactin, while having no effect on the third *B. velezensis* polyketide biosynthesis pathway, which produces bacillaene. Our data also demonstrated that complementation of *loaP* led to increased production of these secondary metabolite synthesis enzymes. These data suggest that all determinants for LoaP regulation are contained within the leader regions of the difficidin and macrolactin synthesis operons. We then purified *B. subtilis* RNA polymerase and the housekeeping sigma factor, as well as the elongation factors NusA, NusG, and LoaP, and reconstituted transcription elongation across the *dfn* leader region *in vitro*. These assays identified both a transcription pause site and a large terminator region within the *dfn* leader, with the terminator site being significantly enhanced by NusA. More importantly, we demonstrated that LoaP binds to a small RNA hairpin, potentially as part of a recruitment mechanism, and promotes readthrough of the terminator region *in vitro* by interfering with NusA activity. These data show that LoaP alone can associate with the TEC to promote antitermination, indicating that the LoaP antitermination complex requires only the LoaP-hairpin ribonucleoprotein complex, and that additional factors, including a leading ribosome, are not necessary. Furthermore, disruption of the LoaP-hairpin ribonucleoprotein complex in the native *B. velezensis* host resulted in significantly decreased production of difficidin and macrolactin antibiotics. Together, these findings reveal the key molecular requirements for LoaP antitermination, which are broadly relevant to the overall class of LoaP regulatory proteins. This knowledge will aid in the design of new synthetic biology tools that can be used to improve the heterologous expression of natural products.

## RESULTS

### LoaP is selectively responsible for the production of polyketide secondary metabolites **in** *Bacillus velezensis*

Genes encoding LoaP are often located adjacent to biosynthetic operons responsible for production of secondary metabolites or polysaccharides. *B. velezensis*, the organism in which *loaP* was first investigated (54), possesses three known polyketide synthesis (PKS) operons, *dfn*, *mln*, and *bae*, which are responsible for the production of the antibiotics difficidin, macrolactin, and bacillaene, respectively (61). In a previous study (54), transcriptomic data showed a moderate potential increase in transcript abundance of *dfn* and *mln* gene clusters following *loaP* induction, suggesting that LoaP may directly or indirectly regulate PKS expression in *B. velezensis*.

In this study, we directly investigated the LoaP regulon using proteomic analysis. We compared four strains: wild type, Δ*loaP*, a *loaP* complementation strain, and an “overexpression” strain that contains the native *loaP* gene as well as an IPTG-inducible, second copy of *loaP*. Cells were grown to mid-exponential growth phase before IPTG was incubated with the cultures for 2 hours. Cells were then collected for trypsin digestion and mass spectrometry analysis to identify proteins that showed significant changes in abundance.

**Fig 1:**
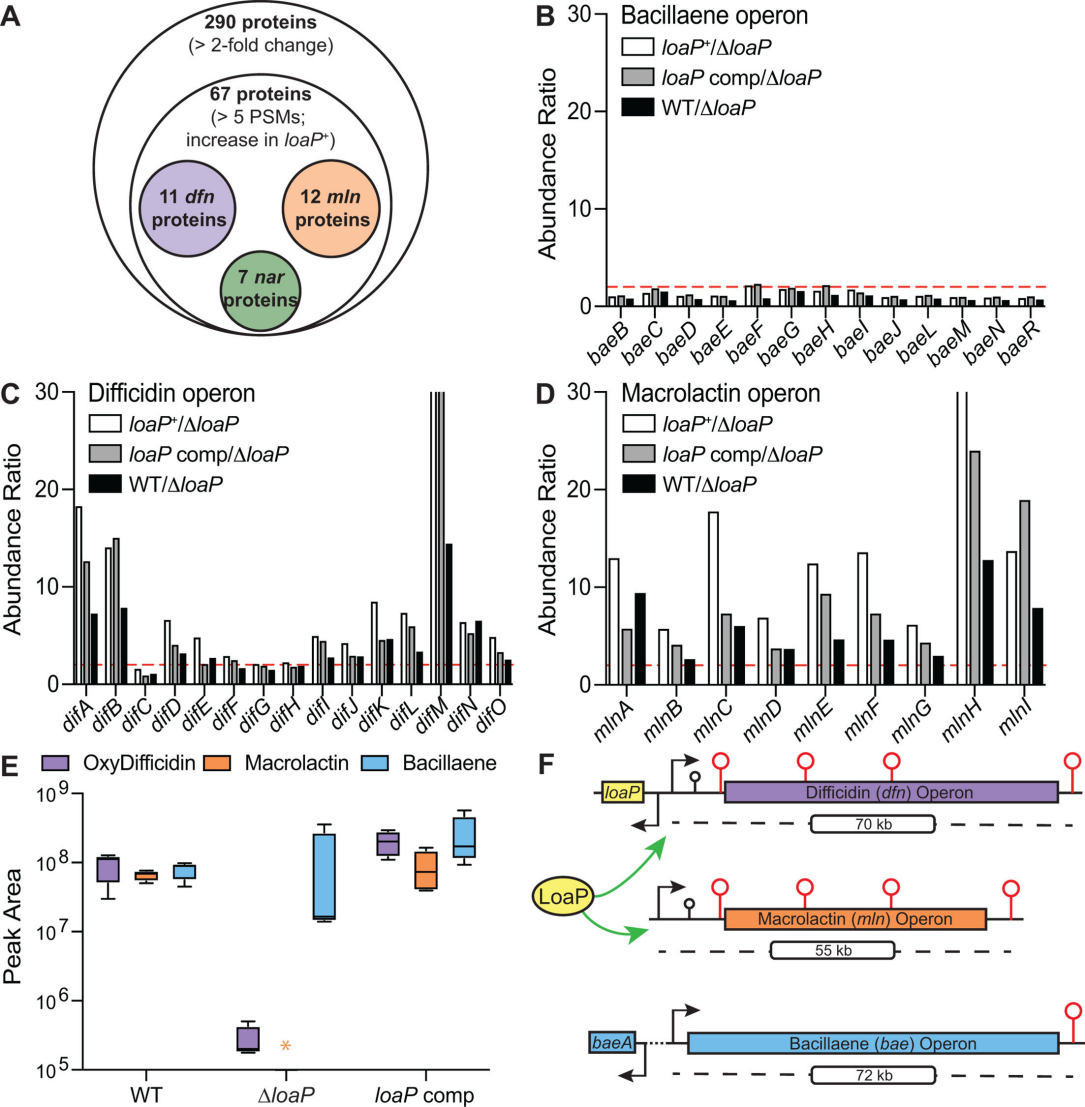
LoaP is selectively responsible for the production of polyketide secondary metabolites in *Bacillus velezensis.* (**A**) Venn diagram showing number of proteins that are altered in response to the expression of LoaP. The outer circle indicates the total number of proteins showing twofold or greater change in abundance, and the inner circle indicates the number of proteins that exceed a minimum threshold detection value and that show increased abundance for both the *loaP* complementation strain and the “overexpression” strain (“loaP*”). (**B–D**) Abundance ratios relative to a Δ*loaP* background for proteins corresponding to the bacillaene (**B**), difficidin (**C**), or macrolactin (**D**) gene clusters determined by mass spectrometry. LoaP overexpression strain denoted “*loaP,^+^*” LoaP complementation strain denoted “*loaP comp.*” Twofold change in abundance threshold represented by the red dotted line. (**E**) Metabolic detection of oxydifficidin, macrolactin, and bacillaene in *B. velezensis* determined by mass spectrometry. An orange asterisk denotes the absence of macrolactin in a Δ*loaP* background. Statistical significance was determined following unpaired t tests. One asterisk denotes a *P*-value < 0.05; two asterisksdenote a *P*-value < 0.01. (**F**) Schematic of LoaP-targeted operons. Green arrows identify gene clusters regulated by LoaP. Black stem loops represent the locations of UNCG tetraloop hairpins. Red stem loops represent the locations of intrinsic terminator hairpins.

A total of 290 proteins showed a greater than twofold increase in abundance in the *loaP* complementation strain as compared to the Δ*loaP* strain (Fig. S1). Of these candidates, 67 proteins exhibited at least five peptide spectral matches and also showed an increase in the *loaP* overexpression strain compared to Δ*loaP* (Fig. 1A). Approximately one-third of these LoaP-upregulated proteins (23 total) were derived from genes located within two of the polyketide synthase gene clusters. Specifically, 11 and 12 proteins were encoded by the difficidin and macrolactin PKS pathways, respectively (Fig. 1C and D). However, bacillaene biosynthetic proteins were not influenced by the presence or absence of the *loaP* gene.

Polyketide synthases are unusually large proteins with a high degree of sequence similarity between one another. This suggests that some of the peptides identified by our proteomic analyses may exhibit redundancy between different polyketide synthases, impacting the reported abundance values of similar proteins. Therefore, we analyzed a truly unique peptide for each gene located throughout the difficidin, macrolactin, and bacillaene biosynthesis gene clusters, which again revealed LoaP-dependent expression of the *dfn* and *mln* genes, but not of the *bae* gene cluster (Fig. S2).

**Fig 2:**
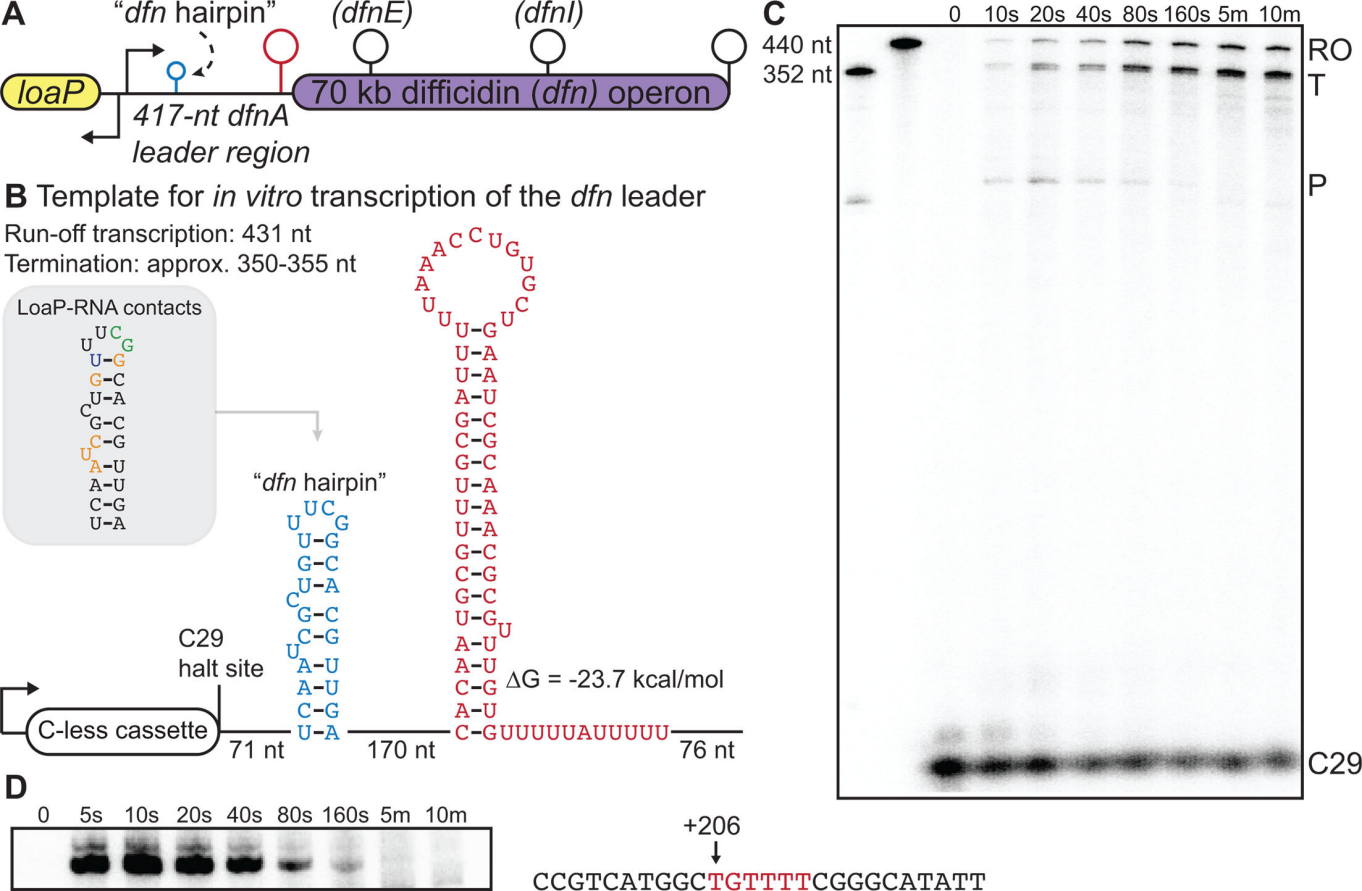
*In vitro* transcription of the *dfn* 5′ leader region. (**A**) Schematic of the *dfn* gene cluster. The *dfn* hairpin is represented in blue, the *dfnA* terminator hairpin in red, and additional *dfn* terminators as black stem loops. (**B**) Features of the DNA template of the *dfn* leader region that was used for *in vitro* synchronized transcription assays. The *dfn* hairpin is represented in blue, and the *dfnA* terminator hairpin in red. Nucleotide lengths between elements and of termination and runoff products are specified. Inset: LoaP-RNA contacts are specified as interaction by the LoaP-NTD (purple), LoaP-CTD (orange), or both terminal domains (green). (**C**) Urea-PAGE of a synchronized transcription reaction on the *dfn* UTR template. Reactions were incubated at 37°C for 15 minutes prior to the addition of NTPs (200 µM), after which aliquots were removed at the specified time points and quenched in stop buffer. Size markers transcribed from PCR products denote termination (T), runoff (RO), and pause (P) products. The C29 halt site is indicated. (**D**) RNAP*_Bs_* pause site (shown in red) resolved via urea-PAGE (left) and characterized via size markers and dideoxy sequencing ladders (not shown).

To directly investigate whether the proteomic data represented meaningful outcomes for production of the polyketide antibiotics, we used mass spectrometry to quantify the relative abundance of the three metabolites (difficidin, macrolactin, and bacillaene) for each of the *loaP* expression strains. This demonstrated that the production of difficidin and macrolactin was entirely dependent on LoaP, whereas bacillaene was produced irrespective of LoaP (Fig. 1E). While prior transcriptomic data have shown that LoaP triggers modest changes in transcript abundance of the *dfn* and *mln* operons (54), the data herein demonstrate that the presence of LoaP is absolutely required for natural product formation; therefore, LoaP regulation is meaningful at the metabolomic level.

Some of the remaining proteins that were enhanced by LoaP may participate in iron and oxidative stress responses, while seven LoaP-elevated proteins are in an operon (*nar*) encoding nitrate/nitrite reductase. This operon also encodes a putative transcriptional regulator enhanced by LoaP. However, manual examination of the genes encoding these proteins did not reveal any known determinants of LoaP antitermination, making it difficult to determine whether these genes were direct or indirect targets of LoaP regulation.

### *In vitro* transcription of the *dfnA* leader region using purified components

We speculate that LoaP relies upon a specific set of molecular determinants that recruit LoaP and orchestrate the molecular mechanism of antitermination. To investigate these molecular requirements, we examined transcription of the unusually long *dfn* leader region *in vitro*. The *Bacillus velezensis* genome is strikingly similar to *Bacillus subtilis*, which is consistent with its former assignment as a subspecies of *B. subtilis* (54, 62)*.* In fact, the transcription factors σ70, NusA, and NusG share 99%, 96%, and 99% sequence identity, respectively, between these organisms, while subunits of the RNAP apoenzyme are 98%–100% identical. Therefore, we reasoned that the *B. subtilis* transcription complex would sufficiently resemble that of *B. velezensis* to allow the formation of the LoaP antitermination complex. To this end, we purified an affinity-tagged *B. subtilis* RNA polymerase from a *nusG*-deficient strain. We also purified NusG and NusA. DNA templates were prepared, wherein the *dfn* promoter was swapped with a constitutive promoter that lacked a CTP incorporation site until position 29 (63). By withholding CTP in the initiation nucleotide mixture, halted complexes were formed at position 29 (Fig. 2A through C), which, by the addition of the missing nucleotide, could be released as synchronized transcription elongation complexes (64). Using this assay, we observed three major products across the length of a DNA template encompassing the 417 nucleotide *dfn* leader region: a pause site, a termination region, and a band corresponding to run-off transcription (Fig. 2C and D). The *dfnA* terminator appeared as two bands that were not adequately resolved during standard urea PAGE and are herein referred to as the *dfnA* “terminator region.”

### NusA enhances the termination efficiency of the *dfnA* intrinsic terminator *in vitro*

**Fig 3:**
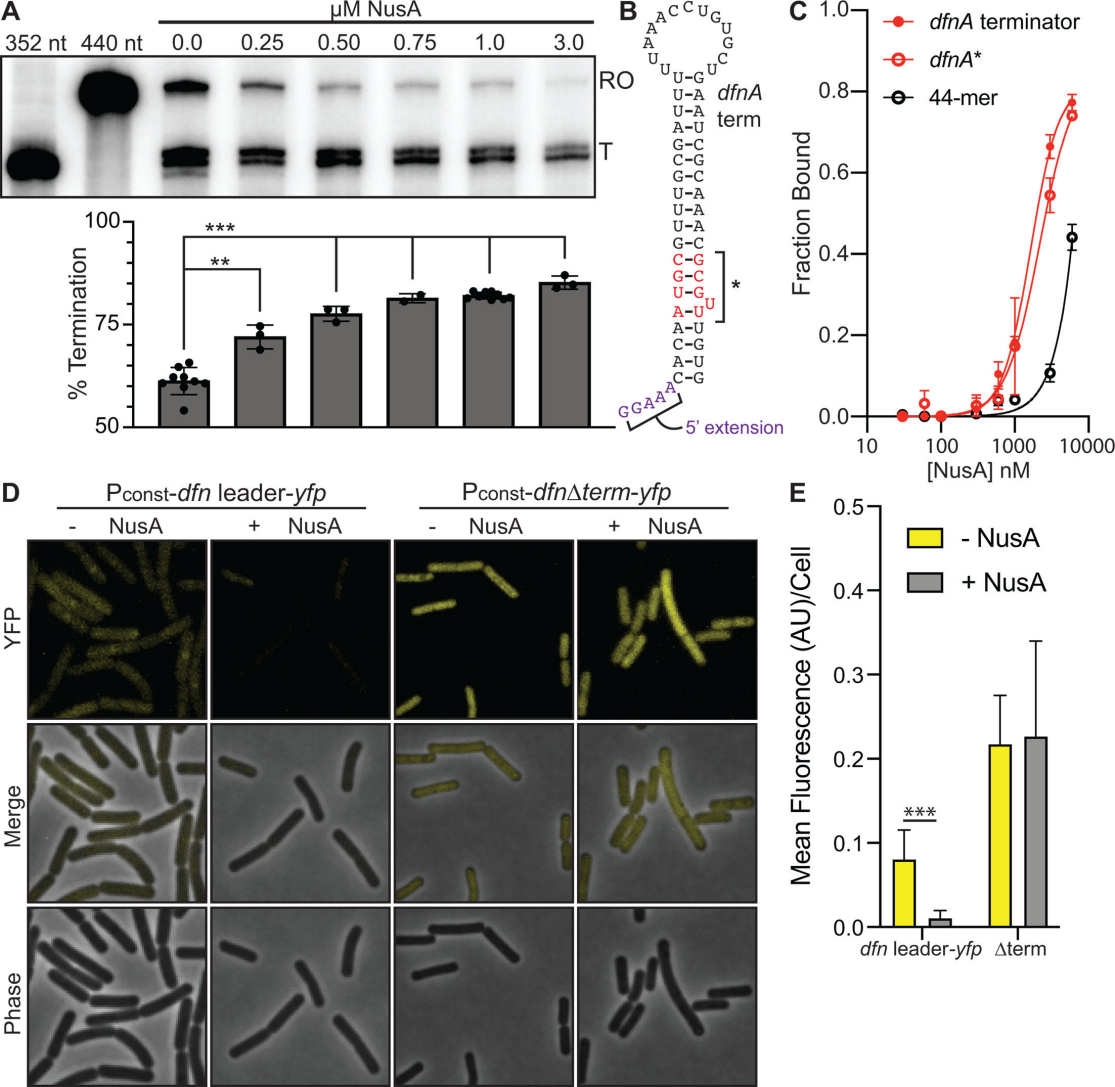
NusA enhances termination within the *dfn* leader region. (**A**) Top: *in vitro* synchronized transcriptions of the *dfn* UTR template with the addition of NusA at the specified concentrations. Halted reactions were incubated at 37°C for 15 minutes prior to the addition of NTPs (200 µM), then quenched in stop buffer after 5 minutes. Size markers transcribed from PCR products denote termination (T) and runoff (RO) products. Bottom: quantification of transcription reactions shown in panel **A**, measuring termination products as a normalized ratio of overall lane intensity. Statistical significance was determined following unpaired t tests. Two asterisks denote a *P*-value < 0.01. Three asterisks denote a *P*-value < 0.001. (**B**) Schematic of the *dfnA* terminator. Residues in red indicate those that were removed from a dfnA* mutant terminator. Residues in purple indicate non-native sequence added to the 5′ end for radiolabeling purposes. (**C**) Equilibrium binding curves obtained via DRaCALA wherein increasing amounts of NusA were combined with radiolabeled synthetic RNAs: *dfnA* terminator (red), a mutant *dfnA* terminator (red open), or a randomized, unstructured 44-mer (black open). The normalized fraction bound is shown with error bars representing the standard deviation from the mean. Binding affinities were calculated from at least three experimental replicates. (**D**) Representative microscopy images of *B. subtilis* expressing either a constitutive P_const_-*dfn* leader-*yfp* reporter or P_const_-*dfn* leader Δterm-*yfp* reporter integrated into the nonessential *amyE* locus, as well as a xylose-inducible copy of *nusA* on a multicopy plasmid. (**E**) Bar graph representing the average fluorescence intensity per cell measured from panel **D**). Statistical significance was determined following unpaired t tests. Three asterisks denote a *P*-value < 0.001.

NusA, an essential protein in *B. subtilis* (65), has been shown to promote termination at intrinsic terminator sites, both *in vivo* and *in vitro,* by enhancing TEC pausing and modulating the RNAP exit tunnel to assist the formation of terminator hairpins (10, 66–68). We added *Bv*NusA into transcription reactions to investigate whether it affects the *dfn* leader. The addition of at least 750 nM *Bv*NusA led to a marked increase in *dfnA* termination efficiency (Fig. 3A). Given that the cytoplasmic concentration of *Bs*NusA has been determined to be approximately 1–4 μM (69), we suspect these results to be physiologically relevant.

NusA forms contacts with the β-flap of RNAP, near the RNA exit tunnel, and TEC-associated *Ec*NusA exhibits RNA-binding activity (70, 71). Therefore, we tested whether purified NusA associates directly with the *dfnA* terminator *in vitro* using a modified filter-binding assay (DRaCALA) (72–74). For this experiment, a radiolabeled RNA representative of the *dfnA* terminator sequence was mixed with increasing concentrations of purified *Bv*NusA, spotted onto nitrocellulose membranes, and then imaged and quantified. In this assay, unbound RNA radially diffuses further than protein-bound RNA, thereby permitting the calculation of the bound fraction. In parallel with the *dfnA* terminator, we also tested a weakened terminator (dfnA*), which lacked several G:C bonds within the terminator stem, and a random unstructured 44-mer. This revealed that NusA bound to all RNA substrates with an equilibrium binding affinity in the low micromolar range (Fig. 3B and C), suggesting that NusA did not exhibit specificity for the terminator sequence. From this, we conclude that *B. subtilis* NusA exhibits nonspecific but detectable RNA-binding affinity *in vitro*.

### NusA affects *dfnA* termination *in vivo*

To assess the influence of NusA *in vivo*, *nusA* was cloned under xylose-inducible control onto a multicopy plasmid, and the *dfn* leader region was transcriptionally fused to *yfp* and integrated single copy into the *B. subtilis* genome at a nonessential locus. Upon induction of *nusA*, the fluorescence of individual cells decreased significantly (Fig. 3D and E). In contrast, NusA had no effect on a reporter system that lacked the *dfn* leader terminator region. Our data together indicate that NusA specifically enhances the formation of the terminator hairpin preceding the first gene in the *dfn* gene cluster, operating as a NusA-dependent, initial regulatory checkpoint for *dfn* expression.

### LoaP antagonizes NusA-mediated intrinsic termination *in vitro*

**Fig 4:**
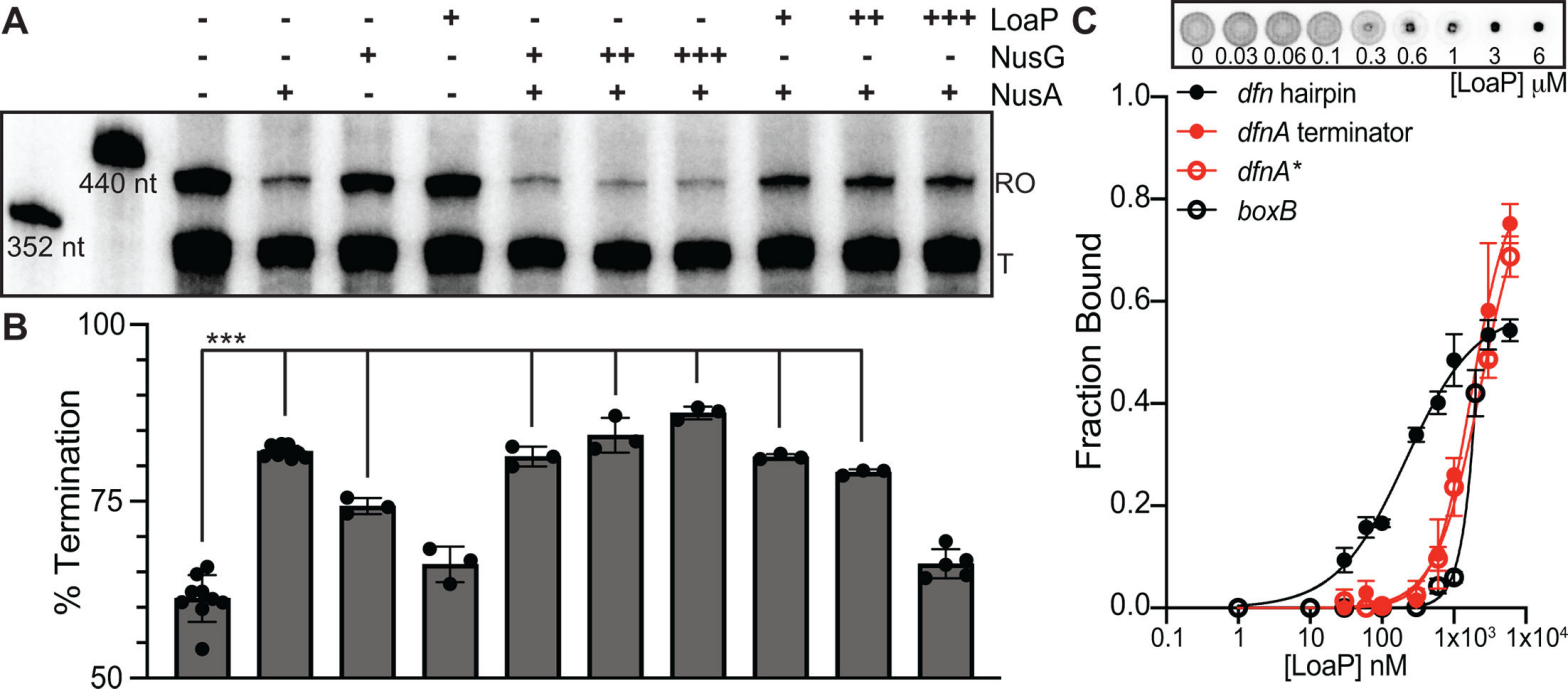
LoaP antagonizes NusA-mediated intrinsic termination *in vitro.* (**A**) *In vitro* synchronized transcriptions of the *dfn* UTR template were incubated with NusA, NusG, and/or LoaP indicated. NusA was added to a final concentration of 1 µM. For NusG and LoaP, final concentrations of 0.1, 0.5, and 1 µM are indicated by +, ++, and +++, respectively. Reactions were incubated at 37°C for 15 minutes prior to the addition of NTPs (200 µM), then quenched in stop buffer after 5 minutes before being resolved by denaturing PAGE; a representative image is shown. (**B**) Bands corresponding to termination products were quantified as a normalized ratio of overall lane intensity. Statistical significance was determined following unpaired t tests. Three asterisks denote a *P*-value < 0.001. Each bar corresponds to the representative data shown in panel **A.** (**C**) Equilibrium binding curves obtained via DRaCALA, wherein increasing amounts of LoaP were combined with radiolabeled synthetic RNAs: *dfn* hairpin (black), *dfnA* terminator (red), a mutant *dfnA* terminator (red open) or the *boxB* hairpin (black open). The normalized fraction bound is shown with error bars representing the standard deviation from the mean. Binding affinities were calculated from at least three experimental replicates.

In previously characterized *E. coli* antitermination mechanisms, it was found that additional elongation factors bound to the TEC to reposition NusA away from the exit tunnel, thereby decreasing termination efficiency (31, 32, 38). To test whether the presence of LoaP and elongation factors could promote antitermination *in vitro*, we conducted synchronized transcription assays of the *dfn* leader region containing physiological amounts of *Bv*NusA (1 µM) and *Bv*LoaP at concentrations ranging from 0 to 1 µM (Fig. 4A). We observed a significant decrease in termination efficiency when 1 µM NusA was combined with 100 nM LoaP (10:1) or higher. Specifically, the presence of LoaP reverted the percentage of terminated transcripts to near that observed on the template alone (~60%). In contrast, the presence of NusG had no effect on termination in the presence or absence of NusA. Additionally, *in vitro* binding experiments assaying for direct interactions between LoaP and the *dfnA* terminator revealed only nonspecific RNA-binding activity by LoaP (Fig. 4B). Thus, LoaP, without the aid of ribosomes or additional elongation factors, can trigger antitermination activity by exerting a direct influence on NusA.

### LoaP antitermination activity is dependent on an RNA hairpin located within the *dfnA* leader region

**Fig 5:**
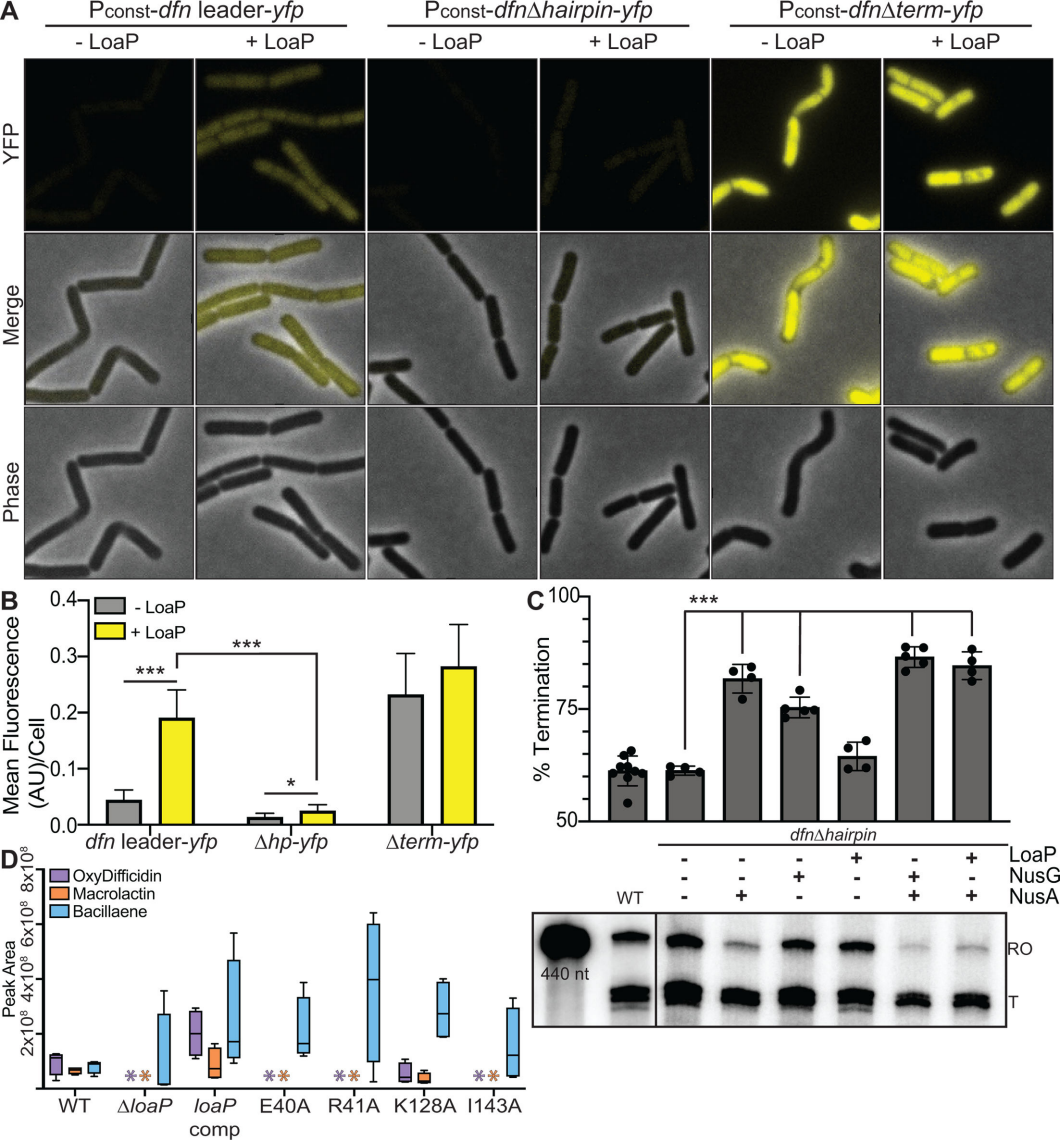
LoaP antitermination activity is dependent on an RNA hairpin located within the *dfnA* leader region. (**A**) Representative microscopy images of *B. subtilis* expressing either a constitutive P_const_-*dfn* leader-*yfp* reporter, a P_const_-*dfn* leader Δhairpin-*yfp* reporter, or a P_const_-*dfn* leader Δterm-*yfp* reporter, each integrated into the nonessential *amyE* locus. Each strain also contained a xylose-inducible copy of *loaP* integrated into the nonessential *thrC* locus. (**B**) Bar graph representing the average fluorescence intensity per cell measured from panel **A**. Statistical significance was determined following unpaired t tests. One asterisk denotes a *P*-value < 0.05. Three asterisks denote a *P*-value < 0.001. (**C**) Bottom: representative *in vitro* synchronized transcriptions of *dfn* UTR template with the addition of NusA, NusG, and/or LoaP (each at 1 µM final), as indicated. Reactions were incubated at 37°C for 15 minutes prior to the addition of NTPs (200 µM), then quenched in stop buffer after 5 minutes before being resolved by denaturing PAGE. Top: quantification of transcription reactions measuring termination products as a normalized ratio of overall lane intensity. Statistical significance was determined following unpaired *t* tests. Three asterisks denote a *P*-value < 0.001. (**D**) Metabolic detection of oxydifficidin, macrolactin, and bacillaene in *B. velezensis*, determined by mass spectrometry. Orange and purple asterisks denote the absence of difficidin or macrolactin, respectively, in a Δ*loaP* background as well as strains expressing LoaP mutants incapable of recognizing the *dfn* hairpin (E40A, R41A, and I143A).

A previous study revealed that a characteristic UNCG-type RNA hairpin (Fig. 2B) is present in both *dfn* and *mln* leader regions and is likely to play an important role in antitermination *in vivo* (53). It was also shown that LoaP proteins from several bacterial species could bind the UNCG hairpin from the *B. velezensis dfn* leader region (“*dfn* hairpin”) with sub-micromolar equilibrium-binding affinity (53). More recently, the high-resolution three-dimensional structure of the LoaP-RNA ribonucleoprotein complex was determined using X-ray crystallography (55). However, the role of this RNA element is unknown, and its general importance has not yet been established. To investigate whether the hairpin is specifically important for antitermination activity *in vivo*, we quantified single-cell fluorescence for a *B. subtilis* strain that contained a transcriptional reporter fusion, wherein *yfp* was placed downstream of the *dfn* leader region. This strain also contained a xylose-inducible *B. velezensis loaP* gene integrated single copy into the genome. Induction of *Bv*LoaP led to a significant increase in YFP signal for a wild-type *dfn* leader region; however, the LoaP-dependent increase in YFP signal was lost after deletion of the hairpin sequence (Fig. 5A and B). Therefore, the hairpin sequence is required for antitermination activity *in vivo*.

To test whether the hairpin affected LoaP antitermination activity *in vitro*, DNA templates specifically lacking the hairpin sequence were used for synchronized transcription assays. Halted TECs were incubated on this template with either NusA alone or a combination of NusA and LoaP. Although the wild-type template showed a LoaP-dependent decrease in NusA-mediated termination (Fig. 4), deletion of the hairpin sequence abrogated this effect (Fig. 5C). After deletion of the hairpin, NusA promoted transcription termination, regardless of the presence or absence of LoaP.

The functional outcome of LoaP antitermination is, in theory, the production of the difficidin and macrolactin antibiotics. Therefore, to investigate whether the LoaP-hairpin interaction is functionally meaningful, beyond proof-in-principle reporter fusions, we tested whether LoaP proteins that contained site-directed mutations of RNA-binding residues would lead to alterations in the synthesis of difficidin and macrolactin. Specifically, mutated *loaP* genes were integrated into a *loaP* deletion strain, and all three polyketide metabolites were quantified by mass spectrometry after the induction of *loaP* expression (Fig. 5D). Each of the site-directed mutations affecting residues involved in RNA-binding activity led to a significant decrease in difficidin and macrolactin levels. As a negative control, K128 was also mutated, as alteration of this position does not affect RNA-binding activity. Together, these data demonstrate that the hairpin element is a critically important determinant of LoaP antitermination and is specifically required for LoaP to interfere with NusA activity.

### A transcriptional pause within the *dfnA* leader region moderately affects antitermination activity *in vivo*

**Fig 6:**
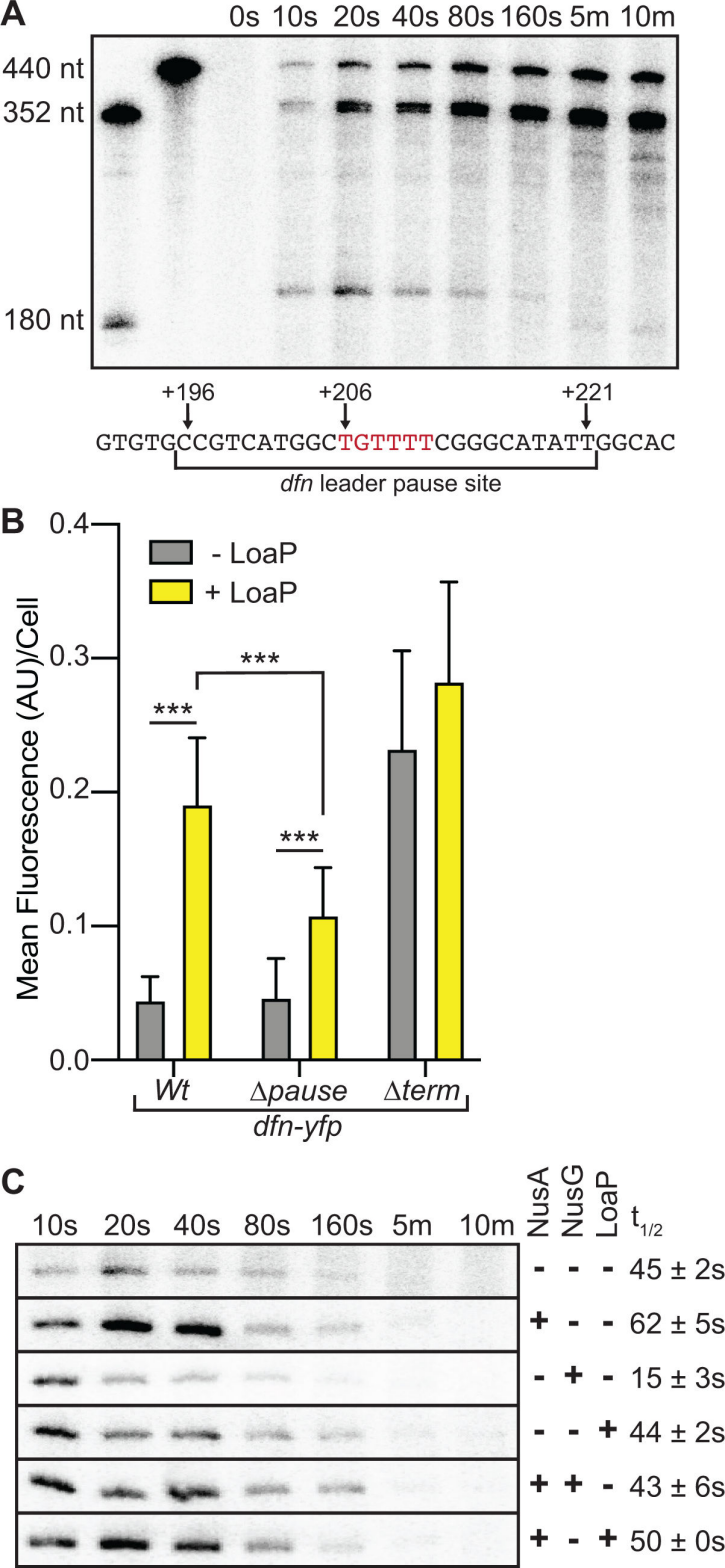
A transcriptional pause within the *dfnA* leader region moderately affects antitermination activity *in vivo*. (**A**) Location of a *Bs*RNAP-specific pause in the *dfn* leader region. Top: representative *in vitro* synchronized transcription of the *dfn* leader region. Reactions were incubated at 37°C for 15 minutes prior to the addition of NTPs (200 µM), after which aliquots were removed at the specified time points and quenched in loading buffer. Size markers were transcribed from PCR products. Bottom: schematic of the *dfn* leader pause site with transcript positions specified. (**B**) Bar graph representing the average fluorescence intensity per cell measured from microscopy of *B. subtilis* expressing a constitutive P_const_-*dfn* leader-*yfp* reporter, a P_const_-*dfn* leader Δpause-*yfp* reporter, or a P_const_-*dfn* leader Δterm-*yfp* reporter, with each integrated into the nonessential *amyE* locus, as well as a xylose-inducible copy of *loaP* integrated into the nonessential *thrC* locus. Statistical significance was determined following unpaired t tests. Three asterisks denote a *P*-value < 0.001. (**C**) *dfn* pause site resolved via urea-PAGE in the presence of NusA, NusG, and/or LoaP, as specified. Reactions were incubated at 37°C for 15 minutes prior to the addition of NTPs (200 µM), after which aliquots were removed at the specified time points and quenched in loading buffer. Pause half-lives (*t*_1/2_) were determined via nonlinear regression analysis.

Synchronized transcription reactions of the *dfn* leader region differed modestly when the reactions contained either *B. subtilis* or *E. coli* RNAP. Specifically, while bands corresponding to termination and run-off transcription were observed with both polymerase complexes, *Ec*RNAP did not generate a pause site that was observed with *Bs*RNAP (Fig. S3). This was not entirely unexpected, as the two polymerases have been shown to differ in their recognition of elongation regulatory signals (75). We mapped the approximate location of the *Bs*RNAP pause site through a combination of size markers and a dideoxy sequencing ladder and found that the pause site resides approximately half of the distance between the *dfn* leader hairpin and the terminator region (Fig. 2C and D; Fig. 6A). Because a strong transcriptional pause is essential for the recruitment of RfaH, we investigated whether the *dfn* leader pause site is also important for LoaP activity *in vivo*. Specifically, we removed the pause site and the surrounding sequence from the *dfn* leader-*yfp* reporter system. When *loaP* was left uninduced, there was no change in readthrough activity compared to the full-length *dfn* leader reporter. However, induction of *loaP* in the absence of the pause sequence resulted in a moderate reduction of antitermination activity when compared to the full-length *dfn* leader control (Fig. 6B).

To test whether the pause site was influenced by transcription factors, we added purified NusA, NusG, and LoaP, either individually or in combination. The *dfn* pause was observed in the absence of purified proteins, suggesting it does not strictly require additional factors. The addition of NusA increased the half-life of the pause site, whereas the addition of NusG decreased the half-life (Fig. 6C). These data add to previously published findings showing these elongation factors can generally affect *B. subtilis* TEC pausing (14–16, 68, 76)*.* Unlike its NusG counterpart, LoaP alone did not alter the pause half-life. However, when NusA and LoaP were added together, the NusA enhancement was reduced overall (Fig. 6C), suggesting that LoaP can antagonize NusA-mediated pausing.

Additionally, while deletion of the pause site did seemingly hinder LoaP antitermination activity *in vitro* (Fig. S4), this effect is much more striking *in vivo*. It is possible that the *in vitro* transcription assay, which relies on a single, synchronized TEC per template, incompletely reflects the transcription elongation environment observed *in vivo*. For example, if the pause site functions *in vivo* to assist in the recruitment of either NusA or LoaP, this requirement may be reduced or eliminated in a synchronized transcription assay format, where saturating levels of proteins are added during the formation of the halted complex. Taken together, these data suggest that the pause site located within the *dfn* leader is not strictly required for antitermination activity but may significantly enhance it.

### LoaP promotes readthrough at additional *dfn* terminators

**Fig 7:**
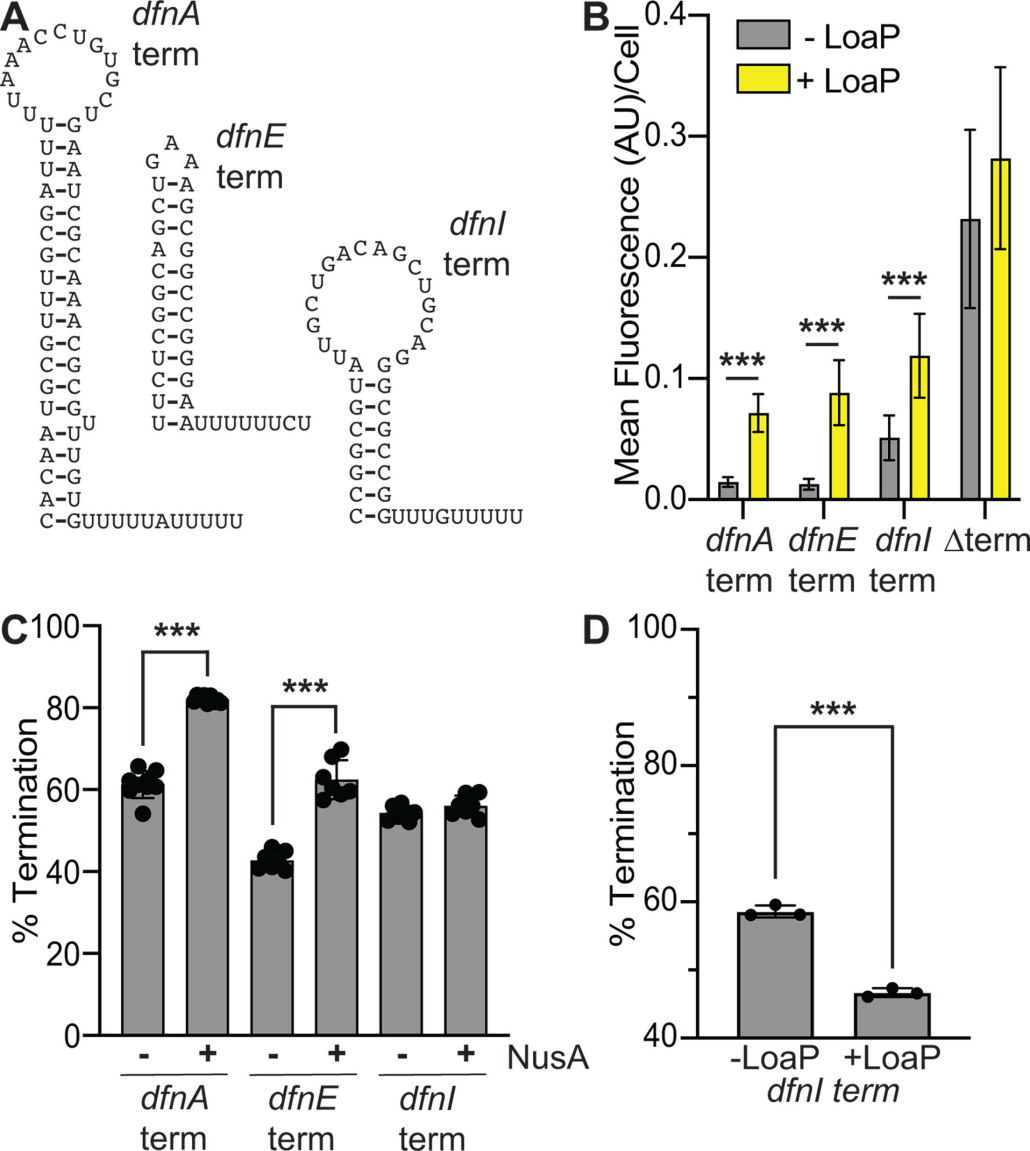
LoaP enacts readthrough at additional *dfn* terminators. (**A**) Schematic of the intrinsic terminator hairpins present in the *dfn* gene cluster. (**B**) Bar graph representing the average fluorescence intensity per cell measured from *B. subtilis* expressing a constitutive P_const_-*dfn* leader-*yfp* reporter containing the terminator sequence specified directly upstream of *yfp*, as well as a xylose-inducible copy of *loaP* integrated into the nonessential *thrC* locus. Statistical significance was determined following unpaired t tests. Three asterisks denote a *P*-value < 0.001. (**C**) Quantification of synchronized transcription reactions using a DNA template representative of the *dfn* leader containing the terminator sequence specified. NusA was added to a final concentration of 1 µM when indicated. Reactions were incubated at 37°C for 15 minutes prior to the addition of NTPs (200 µM), then quenched in stop buffer after 5 minutes. Termination products are measured as a normalized ratio of overall lane intensity. Statistical significance was determined following unpaired t tests. Three asterisks denote a *P*-value < 0.001. (**D**) Quantification of synchronized transcription reactions using a DNA template representative of the *dfn* leader containing the *dfnI* terminator sequence. LoaP was added to a final concentration of 1 µM when indicated. Reactions were incubated at 37°C for 15 minutes prior to the addition of NTPs (200 µM), then quenched in stop buffer after 5 minutes. Termination products are measured as a normalized ratio of overall lane intensity. Statistical significance was determined following unpaired t tests. Three asterisks denote a *P*-value < 0.001.

Prior transcriptomic data (54) suggested that LoaP promoted readthrough of intrinsic terminators located within coding regions of the *dfn* gene cluster. To characterize this antitermination activity *in vivo*, these terminator sequences (*dfnE* and *dfnI*) were substituted in place of the *dfnA* terminator hairpin in the aforementioned *dfn* leader-*yfp* reporter construct. LoaP increased YFP for each reporter construct, showing readthrough at each of the intrinsic terminators present in the *dfn* gene cluster*—dfnA*, *dfnE,* and *dfnI* (Fig. 7A and B). The readthrough effect is notably weaker at the final terminator of the gene cluster, *dfnI*, which, although it requires the *dfn* hairpin for readthrough activity (Fig. S5), permits higher background fluorescence in the absence of LoaP. We then introduced these terminator hairpins into DNA templates for *in vitro* transcription reactions. This revealed a NusA-dependent increase in termination efficiency for the *dfnA* and *dfnE* terminators, but NusA-independent termination for templates possessing the *dfnI* terminator (Fig. 7C). Interestingly, the addition of LoaP alone led to a moderate but reproducible decrease in the termination efficiency of the *dfnI* terminator *in vitro* (Fig. 7D). Taken together, these data suggest that, while LoaP generally promotes readthrough at NusA-dependent termination sites, it may also act alone in promoting antitermination activity at NusA-independent termination sites.

### Deletion of a sequence upstream of the *dfnA* hairpin affects antitermination activity *in vivo*

*Ec*NusG and *Ec*RfaH are both recruited to the TEC via their N-terminal NGN domain (11, 40), which situates the protein immediately adjacent to the non-template DNA sequence. Indeed, RfaH is selectively targeted to operons that contain a characteristic non-template DNA sequence called *ops*, to which RfaH directly binds (40, 56). Similarly, it has been proposed that *B. subtilis* NusG associates with non-template DNA when bound to the TEC (14). In all instances, it is assumed that the NusG NTD-TEC interaction leaves the NusG CTD free to interact with transcription and translation factors. To determine whether LoaP might utilize a non-template DNA recruitment sequence, we modified our *in vivo* reporter system to introduce 10-base pair deletions upstream of the *dfn* hairpin sequence (Fig. S6A and B). The first four deletions had no effect on LoaP antitermination activity *in vivo*. In contrast, deletion of the 10 nucleotides immediately preceding the *dfn* hairpin sequence led to complete loss of antitermination activity. However, inspection of the corresponding region in the *mln* leader did not reveal any obvious sequence similarities. Furthermore, purified LoaP only showed nonspecific, low-affinity interactions with single-stranded DNAs corresponding to these sequence regions (Fig. S6C). Based on these data, we speculate that deletion of this sequence interferes with hairpin function *in vivo* and is not likely to be directly involved in LoaP recruitment. However, our data do not rule out the possibility that a potential interaction between LoaP and non-template DNA sequences might also require an active TEC, or that LoaP may bridge interactions to the upstream DNA duplex, as has been observed for NusG and the NusG specialized paralog UpxY (77).

## DISCUSSION

It has been recently suggested that most Gram-positive bacteria are unlikely to couple transcription with translation (26). Furthermore, the Rho termination factor is absent in some Gram-positive bacteria, such as streptococci, and is nonessential in other bacteria, such as *B. subtilis* (3). These observations directly challenge the assumptions that have been derived from investigations of antitermination mechanisms in Gram-negative bacteria. If the role of Gram-positive antitermination complexes is not to improve coupling to the leading ribosome or to specifically block Rho, then what is their molecular purpose? In lieu of global Rho termination, Gram-positive bacteria appear to rely more heavily on intrinsic terminator hairpin elements. Therefore, it can be assumed that Gram-positive antitermination complexes have evolved molecular strategies that specifically interfere with the termination mechanisms of intrinsic terminator hairpins. Indeed, of the three antitermination mechanisms discovered in Gram-positive bacteria, all have been shown to promote the readthrough of intrinsic terminator hairpins (54, 78, 79). Yet, the molecular basis of their antitermination activity has not been discovered. To address this gap in knowledge, we investigated the antitermination mechanism employed by *Bacillus velezensis* LoaP, a NusG paralog encoded by many Gram-positive bacteria.

In a previous study (54), transcriptomic data suggested that *B. velezensis* LoaP influences two PKS gene clusters. These gene clusters, which produce the secondary metabolites difficidin (*dfn*) and macrolactin (*mln*), are unusually long and adorned with several intrinsic terminator hairpins that likely evolved as an additional layer of regulatory control against the expression of energetically costly operons. Additional analyses revealed a UNCG-tetraloop RNA hairpin element (*dfn*) present in the leader regions of both gene clusters (53). We were unable to locate the hairpin structure within the leader region of the third PKS gene cluster expressed by *B. velezensis*, which is responsible for the production of bacilleane (*bae*)(61). In this study, we used proteomic analyses to search for proteins that were increased upon LoaP expression and that decreased in a *loaP* deletion strain. This revealed that peptides unique to proteins from the *dfn* and *mln* gene clusters were highly dependent on *loaP* expression, with most being completely undetectable in the absence of LoaP. However, this was not the case with peptides unique to the *bae* gene cluster, whose abundance did not change with respect to *loaP* expression. These data demonstrated that *B. velezensis* LoaP acts specifically on the *dfn* and *mln* operons. The fact that both operons contain a nearly identical UNCG-tetraloop hairpin sequence within their 5′ leader regions suggests a mechanistic role for this RNA determinant. Furthermore, each 5′ leader region contained a putative intrinsic terminator hairpin, suggesting that these terminator sites are likely to be key points of regulatory control. This observation also suggests that all molecular determinants required for LoaP antitermination are contained within the leader regions of these target regulons.

We reasoned that a DNA template encompassing the *dfn* leader region could be used to reconstitute LoaP antitermination *in vitro*. Using synchronized transcription reactions, in which only a single TEC could operate on a given DNA template, we found that the *dfn* leader region contained a functional terminator site that was enhanced by the presence of NusA. Concurrent addition of NusG had no effect on NusA-mediated termination; however, the addition of LoaP significantly decreased NusA-mediated termination. From this, we conclude that LoaP’s antitermination activity derives from its ability to directly antagonize NusA. This is unique among all known antitermination mechanisms and is likely to be broadly relevant for the subclass of LoaP regulatory proteins. From our data, we speculate that NusA manipulates the exit tunnel of RNAP to better support the formation of the nascent hairpin, a function that has been previously characterized for *Ec*NusA (9, 10). We also propose that LoaP disrupts NusA function by potentially repositioning NusA away from the exit tunnel, without the need for additional factors.

Earlier binding assays found that LoaPs purified from multiple species all bind the *dfn* UNCG-type hairpin with sub-micromolar affinity and high specificity (53), using both LoaP’s N- and C-terminal domains (55). To investigate the importance of this ribonucleoprotein complex, we deleted the hairpin from a *dfn* leader region*-yfp* reporter fusion and found that it resulted in a loss of antitermination activity as compared to a wild-type *dfn* sequence. Moreover, site-directed mutagenesis of RNA-binding residues (55) resulted in a significant decrease in cellular production of difficidin and macrolactin, demonstrating that the RNA-binding activity of LoaP is essential for antibiotic production *in vivo*.

**Fig 8:**
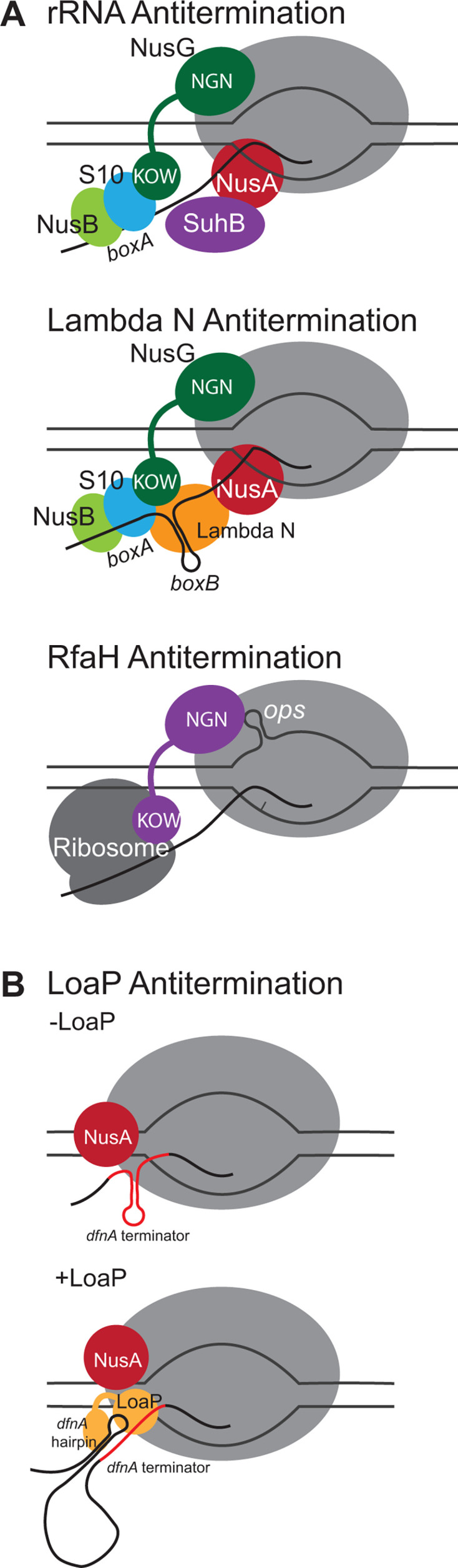
Graphical summary of antitermination mechanisms. (**A**) Summary schematics of previously characterized *E. coli* antitermination mechanisms. Top: rRNA. Middle: λN. Bottom: RfaH. (**B**) Summary schematic of LoaP antitermination. Top: NusA enhances intrinsic termination at *dfnA* in the absence of LoaP. Bottom: LoaP antagonizes NusA-enhanced intrinsic termination.

LoaP is the only known bacterial NusG-like protein that specifically associates with an RNA element. Although high-resolution structural data of the LoaP antitermination complex are needed to unambiguously determine the role of the ribonucleoprotein complex, we speculate that it may play a role in the recruitment of LoaP to the TEC. Both NusG and RfaH rely on their NTDs to interact near or with sequences of the non-template DNA strand (11, 40). More specifically, RfaH takes advantage of an RNAP pause brought about by a DNA hairpin in the non-template strand, *“ops”* (40, 56). Upon forming in the transcription bubble, this sequence stalls the TEC, giving RfaH time to contact its DNA recognition sequence and thereby activating an otherwise autoinhibited RfaH (58). In contrast, the *dfn* hairpin results in no discernible TEC pause, implying that LoaP binds the *dfn* hairpin after it has left the TEC exit tunnel. This, in turn, suggests that the LoaP-hairpin complex associates with the TEC as it actively polymerizes away from the LoaP recruitment site. This might explain why a downstream transcriptional pause site assists LoaP antitermination both *in vivo* and *in vitro*; we speculate that the downstream pausing event gives the LoaP-hairpin complex time to associate with the TEC. Additionally, LoaP interferes with the ability of NusA to lengthen this pause event, further supporting the model that LoaP, by potentially disrupting NusA’s position at the exit tunnel, antagonizes NusA activity as part of the TEC (Fig. 8B). Together, these data reveal fundamental features of the LoaP mechanism and provide key insights into the antitermination strategies used by Gram-positive bacteria. Uncovering the molecular basis of LoaP regulation will aid in the discovery and production of natural products and other secondary metabolites. *loaP* genes are frequently near secondary metabolite biosynthesis operons (54); therefore, it is likely that secondary metabolite pathways are frequent targets of LoaP regulons. Understanding the regulatory mechanism of LoaP may lead to new strategies for boosting the production of unidentified metabolites. In addition, knowledge of the LoaP mechanism may be used to develop novel genetic tools that improve heterologous expression of antibiotics and other natural products.

## MATERIALS AND METHODS

### Strain construction

All strains utilized in this study are summarized in Table S1. To construct *E. coli* strains to overexpress and purify the proteins used in this study, the coding sequences for *loaP_Bv_*, *nusA_Bv_*, *nusG_Bv_*, and *sigA_Bs_* were PCR-amplified from genomic DNA (*B. velezensis* or *B. subtilis*) or purchased from Integrated DNA Technologies (IDT) and cloned via Gibson assembly into a plasmid (pAmr30) containing an IPTG-inducible N-terminal decahistidine tag fused to a bdSENP-cleavable SUMO tag. Plasmids were assembled using standard molecular cloning strategies and transformed into *E. coli* XL10-Gold (Agilent) for plasmid replication. Plasmids were transformed into *E. coli* T7 Express (New England BioLabs) for overexpression. A *B. subtilis* strain lacking *nusG* and possessing a His-tagged RNAP was assembled by transforming strain 1A813 (*B. subtilis* 168, *rpoC*::10×His tag, BGSC) with genomic DNA isolated from strain BKE01010 (*B. subtilis* 168 Δ*nusG::erm*, BGSC). *In vivo* reporter strains were constructed by PCR-amplifying the region corresponding to the *dfn* leader region and subcloning this sequence, followed by *yfp*, into the vector pDG1662, which integrates into the *amyE* nonessential gene locus. Mutations in this leader region were made via site-directed mutagenesis. A plasmid that integrates a xylose-inducible *loaP_Bv_* into the *thrC* nonessential gene locus (pJG108) was used to control LoaP production, whereas a multi-copy, conjugatable plasmid (pEP011) was used as the vector for a xylose-inducible *nusA_Bv_*.

### Protein overexpression and purification

*E. coli* strains harboring T7 expression vectors for 10×His-SUMO-tagged protein sequences were cultured in 2×YT supplemented with 100 µg/mL carbenicillin, shaking, overnight at 37°C. Cultures were diluted into fresh media and grown shaking at 37°C until reaching an OD_600_ ~ 0.4–0.8, at which point cells expressing LoaP*_Bv_* were induced with 1 mM IPTG and grown for an additional 18–20 hours at room temperature, while cells expressing NusA*_Bv_* or NusG*_Bv_* were induced with 0.5 mM IPTG and grown for an additional 3 hours at 37°C. Cells were harvested via centrifugation and resuspended at 12 mL/g wet cell weight in lysis buffer (LoaP lysis: 50 mM Na_2_HPO_4_, pH 7.2, 1 M NaCl, 10 mM EDTA, 1 mM DTT, 1 mM PMSF, 5% glycerol; NusA/NusG lysis: 50 mM Tris-HCl, pH 8, 1 mM DTT, 1 M NaCl, 5% glycerol, 10 mM imidazole, 0.5 mM PMSF). Cells were lysed via sonication before polyethyleneimine was added slowly and mixed with gentle rocking to a final concentration of 0.5% (vol/vol). Any insoluble material and precipitated nucleic acids were removed via centrifugation. Clarified lysates were collected, and a 20–70% ammonium sulfate (wt/vol) cut was performed. Ammonium sulfate pellets were resuspended in 30 mL resuspension buffer (LoaP resuspension: 50 mM Na_2_HPO_4_, pH 7.2, 100 mM NaCl, 0.1 mM EDTA, 5% glycerol; NusA/NusG Resuspension: 50 mM Tris-HCl, pH 8, 0.5 mM DTT, 500 mM NaCl, 5% glycerol, 10 mM imidazole). Supernatants were incubated with cOmplete His-Tag purification resin (Roche) at 4°C for 1 hour, with gentle rocking, before transferring to a gravity column and draining the flowthrough. Each column was washed with 10 column volumes (CV) of the respective resuspension buffer, followed by 10 CV of 50 mM imidazole in resuspension buffer. LoaP was eluted with 5 CV of 400 mM imidazole in Resuspension buffer. NusA and NusG were each eluted with 5 CV of 250 mM imidazole in resuspension buffer. Elutions were transferred to sterile tubes, and MgCl_2_ and DTT were each added to a final concentration of 2 mM. Elutions were then incubated with bdSENP1 protease on ice overnight. Cleaved elutions were dialyzed against IEX-loading buffer (LoaP: 10 mM Na_2_HPO_4_, pH 7.2, 150 mM NaCl, 0.1 mM EDTA, 1 mM DTT, 0.5% glycerol; NusA/NusG: 20 mM Tris-HCl, pH 8, 5% glycerol, 100 mM NaCl, 1 mM DTT) at 4°C for >2 hours. A Mini Strong Ion Exchange Spin Column (Pierce; Cation for LoaP, Anion for NusA/NusG) was equilibrated in the respective IEX-loading buffer and centrifuged at 2,000 × *g* for 5 minutes. Samples were loaded onto the spin columns 400 µL at a time and centrifuged each time. The column was washed twice with 400 µL IEX-loading buffer containing 400 mM NaCl before eluting protein with 400 µL IEX-loading buffer containing 800 mM NaCl. The eluted protein fractions were dialyzed overnight against storage buffer (LoaP: 20 mM Na_2_HPO_4_, pH 7.2, 200 mM NaCl, 1 mM DTT, 0.1 mM EDTA, 10% glycerol; NusA/NusG: 20 mM Tris-HCl, pH 8, 5% glycerol, 100 mM NaCl, 1 mM DTT) at 4°C. Dialyzed elutions were concentrated to at least 100 µM and flash-frozen in liquid nitrogen. Samples were stored in 0.1 mL aliquots at −80°C or at −20°C with 50% (vol/vol) glycerol after thawing.

#### RNAP*_Bs_*

*B. subtilis* Amr64 (Δ*nusG*, 10×His-*rpoC*) was grown at 37°C, with shaking, in LB to OD_600_ = 1.0, then pelleted by centrifugation at 5,000 × *g*, and stored at −80°C. A total of 50 g frozen cell pellet was thawed on ice and resuspended in 50 mL of lysis buffer (40 mM Tris-HCl, pH 8, 5 mM MgCl_2_, 10% glycerol, 1 mM β-ME, 300 mM NaCl, 10 mM imidazole, 1 mM PMSF). Cells were lysed by sonication on ice for six cycles (each 12 s on, 48 s off) at 30% amplitude. Lysates were clarified at 16,000 × *g* for 40 minutes. The clarified lysate was incubated with 1 mL HisPur Ni NTA resin (Thermo Scientific; pre-equilibrated in lysis buffer) on ice, with gentle rocking, for 30 min before transferring to a gravity column and slowly draining the flowthrough (~0.5 mL/min). The column was washed twice with 20 mL of wash buffer (40 mM Tris-HCl, pH 8, 5 mM MgCl_2_, 10% glycerol, 1 mM β-ME, 300 mM NaCl, 20 mM imidazole) and eluted with 3 mL of elution buffer (40 mM Tris-HCl, pH 8, 5 mM MgCl_2_, 10% glycerol, 1 mM β-ME, 100 mM NaCl, and 200 mM imidazole). Eluted protein was dialyzed in 40 mM Tris-HCl, pH 8, 5 mM MgCl_2_, 10% glycerol, 1 mM β-ME, 100 mM NaCl before being concentrated and flash-frozen or stored in 50% glycerol at −80°C.

#### σA*_Bs_*

*B. subtilis* Amr80 (SUMO-6×His*-*σ*A*) was grown at 37°C, with shaking, in LB to OD_600_ = 0.6–0.8 and induced with 0.1 mM IPTG. The cells were moved to room temperature and grown for an additional 16 hours before being pelleted by centrifugation at 5,000 × *g* and stored at −80°C. Frozen pellets were thawed on ice and resuspended in 10 mL/g of lysis buffer (50 mM Tris-HCl, pH 8, 300 mM NaCl, 5% glycerol, 20 mM imidazole, 0.1 mM EDTA). Cells were lysed by sonication on ice for six cycles (each 1 min on, 1 min off) at 40% amplitude. Lysates were clarified at 16,000 × *g* for 15 minutes. The clarified lysate was incubated with 1 mL HisPur Ni NTA resin (Thermo Scientific; pre-equilibrated in lysis buffer) on ice, with gentle rocking, for 2 hours before transferring the gravity column and slowly draining the flowthrough (~0.5 mL/min). The column was washed with 15 mL of lysis buffer, followed by the addition of 10 mL of cleavage buffer (50 mM Tris-HCl, pH 8, 100 mM NaCl, 0.1 mM EDTA, 1 mM MgCl_2_, 1 mM DTT, 5% glycerol) and bdSenp1 protease. The column was incubated overnight, with gentle rocking, at 4°C, and the flowthrough was collected the next day. Cleaved protein was further concentrated before being loaded onto a Mini Strong Anion Exchange Spin Column (Pierce). The spin column was washed twice with 50 mM Tris-HCl, pH 8, 100 mM NaCl, 0.1 mM EDTA, and 5% glycerol, and then eluted with 50 mM Tris-HCl, pH 8, 200 mM NaCl, 0.1 mM EDTA, and 5% glycerol. The eluted protein was further concentrated, and the buffer was exchanged to a final (NaCl) of 100 mM. Aliquots were either flash frozen or stored in 50% glycerol at −80°C.

### Synchronized transcription assays

Transcription elongation complexes were established by combining 100 nM linear DNA templates with 500 nM *B. subtilis* RNAP apoenzyme in transcription buffer (20 mM Tris-HCl, pH 8.0, 40 mM KCl, 5 mM MgCl_2_, and 1 mM β-mercaptoethanol). Transcription reactions were supplemented with 500 nM purified *B. subtilis* sigma factor A (σA) and 100 µM ApG to assist in initiation. These transcription mixtures included ATP and GTP at 5 µM and UTP at 2.5 µM, but lacked CTP. They were incubated for 15 min at 37°C, resulting in elongation complexes being halted on the templates at C29 (Fig. 2C). Elongation was stimulated by the addition of nucleotides (ATP, CTP, GTP, and UTP at 200 µM) and heparin (100 µg/mL) to limit re-initiation of free polymerases. Transcription progress was tracked via ^32^P derived from the incorporation of [α-^32^P]UTP (3,000 Ci/mmol). After 5 min, additional NTPs (Chase) were added (250 µM each). When included, transcription factors NusA, NusG, and LoaP were added during the formation of the halted complex to final concentrations indicated in the figures. Samples were removed in the time frames specified in the figures and quenched in stop buffer (10 M urea, 20 mM EDTA, 45 mM Tris-borate, pH 8.3) prior to phenol extraction. The samples were heated for 2 min at 90°C and resolved by 6% denaturing PAGE containing 1× TBE and 4 M urea. RNA products were visualized and quantified as a normalized ratio of overall lane intensity using a Cytiva Amersham Typhoon PhosphorImaging System, ImageJ Software, and Microsoft Excel. Half-life calculations of the *dfn* leader pause site were performed as previously described (80). All synchronized transcription data were collected from at least three technical replicates.

### Proteomics sample preparation and normalization

To quantify the effect of LoaP on the proteome of *Bacillus velezensis* FZB42, WT, ∆*loaP*, and *loaP* overexpression and complementation strains were prepared. At least four biological replicates of each strain were cultured in 5 mL LB to an OD_600_ of 0.8 at 37°C. Dried protein pellets were resuspended in 500 µL lysis buffer (5% sodium dodecyl sulfate, 50 mM triethylammonium bicarbonate) and sonicated with a Branson probe at 50% amplitude for 25 s, with 5 s on/off cycles. The protein concentration of each sample was assessed using a BCA Protein Assay kit (Pierce) following the manufacturer’s protocol. A total of 300 µg of each sample was used for proteomic digestion. Dithiothreitol (DTT) was added to each aliquot to a final concentration of 0.2 M, followed by incubation at 95°C for 10 min. After cooling to room temperature, iodoacetamide was added to each sample to a final concentration of 0.1 M. Samples were then incubated in the dark at room temperature for 30 min. Formic acid (15%) was added to each sample at a final concentration of 1.5%, and S-Trap buffer (90% methanol, 100 mM triethylammonium bicarbonate) was added to each sample at 6× the total reaction volume. The samples were loaded into S-Trap Mini columns (Protifi), and loading and wash steps were performed according to the manufacturer’s protocol. Finally, 125 µL of 50 mM triethylammonium bicarbonate containing trypsin Lys-C (at a 1:20 enzyme-to-protein ratio; Promega) was added to each S-Trap Micro column filter. The columns were wrapped with parafilm to prevent evaporation and placed in a stationary incubator overnight at 37°C. Elution was performed the following morning according to the manufacturer’s instructions. Eluted peptides were dried and resuspended in water and 30% acetonitrile. Peptide concentrations of each sample were determined using a Colorimetric Peptide Assay (Pierce) following the manufacturer’s protocol. Peptides were dried once more, then resuspended in 5% acetonitrile and 0.1% formic acid in water to a final peptide concentration of 1 µg/µL for analysis via LC/MS/MS.

### Proteomics data acquisition

Each sample was analyzed using a Thermo Fisher Orbitrap Fusion mass spectrometer coupled to a Dionex Ultimate 3000 UHPLC (Thermo Fisher Scientific). Injections of each sample (2 µL) were first pre-concentrated on a reverse-phase trapping column (PepMap 300 µm × 5 mm C18, 100 Å, Thermo Fisher) and then resolved on a 75 µm × 500 mm EASY-Spray column packed with PepMap RSLC C18, 2 µm, 100 Å particles (Thermo Fisher) using a 190 min multistep gradient (0–150 min: 6%–35% B, 150–158 min: 35%–60% B, 158–161 min: 60%–90% B, 161–171 min: 90% B hold, 171–172 min: 90%–96% B, and 172–182 min: 6% B hold). For the gradient, buffer A was 100% H_2_O/0.1% formic acid, and buffer B was 98% acetonitrile/2% H_2_O/0.1% formic acid. MS1 scans were acquired using the Orbitrap at a resolution of 120,000 with a scan range of *m/z* 350–1,500 in profile mode. The top 15 precursors were selected for MS2 data-dependent fragmentation. MS2 spectra were acquired using the ion trap with the scan rate set to “Turbo.” The minimum signal required to trigger a data-dependent scan was 5,000. Monoisotopic precursor selector was set to “On,” and charge state filter was set to include charge states 2–7. High-energy C-trap dissociation (CID) was used to generate MS2 spectra with a collision energy of 65%. The AGC targets were set to 200,000 for MS1 and 10,000 for MS2. The maximum accumulation time for MS1 and MS2 was 50 ms. Dynamic exclusion was set for 70 s with a 10 ppm mass window.

### Proteomics data analysis

Including database discovery, relative abundance plots, cluster map, principal components analysis (PCA) plot, and volcano plot generation, untargeted proteomics data analysis was performed with Thermo Fisher Scientific Proteome Discoverer (ver. 2.5.0.400). A database search was performed against the Uniprot canonical and isoform FASTA (Taxon ID: 1390), downloaded from Uniprot (6 March2023), and searched using Sequest. The search parameters included an MS1 tolerance of 10 ppm and an MS2 tolerance of 0.6 Da. A variable protein N-terminal acetylation was also included in the search, along with oxidation of methionines and static carbamidomethylation of cystines. FDR control was achieved utilizing the Percolator node. Post-translational modification (PTM) localization was performed with IMP-ptmRS. Label-free quantitation was performed using the Minora feature-detection node. Identified proteins from the analysis were then filtered by requiring at least one unique peptide, more than 1 PSM, and Protein FDR at a confidence level (0.01 FDR).

### Metabolomic extraction

Complementation strains encoding *loaP* with amino acid mutations thought to inhibit the binding to the *dfn* hairpin (E40A, R41A, and I143A) were processed for metabolomic analysis. A strain with a LoaP K128A mutation was used as a negative control, as the mutation previously showed no observable change in RNA-binding affinity *in vitro*. Four biological replicates of each strain were grown overnight at 37°C with shaking, in 5 mL cultures of LB. Samples were then sub-cultured in 100 mL of LB and grown at 37°C with shaking to an OD_600_ of 0.5. Xylose was added to the cultures at a final concentration of 0.5% (wt/vol) to induce *loaP* gene expression. The samples were then grown for 2 hours at 37°C with shaking. Supernatants were collected by centrifugation, followed by the addition of 8 g of Amberlite XAD-16 resin. The samples were then shaken overnight at 4°C. The resin was washed with 50 mL of LCMS grade water, followed by elution in 8 mL of LCMS grade methanol. The samples were dried to completion and resuspended in 200 µL of 90:10 H_2_O:ACN with 0.1% FA.

### Metabolomic data acquisition

The metabolomic samples were run on a Thermo Scientific Vanquish HPLC and Q Exactive Plus Hybrid Quadrupole-Orbitrap Mass Spectrometer. 10 µL of sample was injected onto a CORTECS T3 Column (120 Å, 2.7 µm, 2.1 mm × 150 mm) and resolved on a 20 min gradient ranging from 0% B (ACN with 0.1% formic acid) and 100% A (H_2_O with 0.1% formic acid) to 95% B and 5% A. Sample was injected twice for both positive and negative mode acquisitions.

### Metabolomic data analysis

Metabolomic data analysis was initially performed using the FisH scoring metric in Compound Discoverer to obtain *in silico* fragmentation spectra for each analyte of interest. Data were then processed in Skyline with an MS1 ppm tolerance of 5 ppm and 0.02 fragment tolerance. The area under the curve was calculated using a built-in peak-finding tool.

### Differential radial capillary action of ligand assay (DRaCALA)

All RNA and DNA species used in the *in vitro* binding assays are summarized in Table S2 and were purchased from IDT and 5′-radiolabeled with [γ-^32^P]ATP using T4 Polynucleotide Kinase (T4PNK; New England Biolabs). Longer nucleic acid species (>17 bp) were purified using a Zymo RNA Concentrator and Clean-up Kit. Binding reactions were set up in 96-well plates by incubating 2.5 nM radiolabeled RNA with increasing amounts of LoaP in binding buffer (50 mM NaH_2_PO_4_, pH 7.2, 100 mM NaCl, 0.1 mM EDTA, 1 mM MgCl_2_, 100 µg/mL BSA, 25 µg/mL total yeast RNA) for 45 min at room temperature in the dark. 2 µL aliquots were spotted onto nitrocellulose membranes using a fixed replicator pin tool and allowed to air dry for 20 min. Nitrocellulose membranes were exposed to a phosphor screen for 20 min, visualized using an Amersham Typhoon phosphorimager, and quantified using ImageJ and GraphPad Prism software. All binding data were collected from at least three technical replicates.

### Fluorescence microscopy

2×YT medium (containing the appropriate antibiotic) was inoculated from single colonies and incubated at 37°C with shaking, overnight. These cultures were centrifuged at 5,000 × *g* for 5 min and resuspended in fresh medium without antibiotics. Cultures were then diluted to an OD_600_ of 0.1 and incubated at 37°C with shaking until reaching an OD_600_ of 0.5. D-xylose (1% [wt/vo] final) was added to the cultures, and the cells were incubated for an additional 3 hours. The cultures were then centrifuged at 5,000 × *g* for 5 min and resuspended in 1× phosphate-buffered saline. This step was repeated, and aliquots were placed on 1.5% low-melting-point agarose pads and allowed to dry for 10 min before being inverted on a glass coverslip. The cells were imaged at room temperature using a Zeiss Axio-Observer Z1 inverted fluorescence microscope equipped with a YFP filter cube and enclosed in a temperature-controlled chamber. Fluorescence intensity per cell was quantified using ImageJ software. Each mean fluorescence value was determined across a minimum of 30 cells.

## Data Availability

All primary data are deposited on an archived site on the Mendeley Data website and can be found at “Jermain et al 2025 LoaP antagonizes NusA,” Mendeley Data, V1, doi: 10.17632/d3d9wntsf6.1. The mass spectrometry proteomics data have been deposited to the ProteomeXchange Consortium via the PRIDE partner repository with the data set identifier PXD063323.

## SUPPLEMENTAL MATERIAL

Supplemental material mbio.01429-25-s0001.pdf Figures S1-S6 and Tables S1 and S2.

ASM does not own the copyrights to Supplemental Material that may be linked to, or accessed through, an article. The authors have granted ASM a non-exclusive, world-wide license to publish the Supplemental Material files. Please contact the corresponding author directly for reuse.
