# Supplementary material for: Bacillus velezensis LoaP promotes antitermination by antagonizing NusA
Source: mBio. 2025 Oct 28;16(12):e01429-25. doi: 10.1128/mbio.01429-25 (PMC12691694; doi:10.1128/mbio.01429-25)
Supplement: Supplemental material — Figures S1-S6 and Tables S1 and S2. [file mbio.01429-25-s0001.pdf]

Supplementary Materials

Bacillus velezensis LoaP Promotes Antitermination by Antagonizing NusA

Madison D. Jermain[1], Thao Tran[2], Conor C. Jenkins[2], Benjamin H. Nasisi[1], Wade C. Winkler[1,2]

[1]Department of Cell Biology and Molecular Genetics, University of Maryland, College Park, Maryland, 20742, USA

[2]Department of Chemistry and Biochemistry, University of Maryland, College Park, Maryland, 20742, USA

Table of Contents

Pages 2	**Supplemental Figure 1**
Page 3-4	**Supplemental Figure 2**
Page 5	**Supplemental Figure 3**
Page 6	**Supplemental Figure 4**
Page 7	**Supplemental Figure 5**
Page 8	**Supplemental Figure 6**
Page 9	**Supplemental Table 1**
Page 10	**Supplemental Table 2**

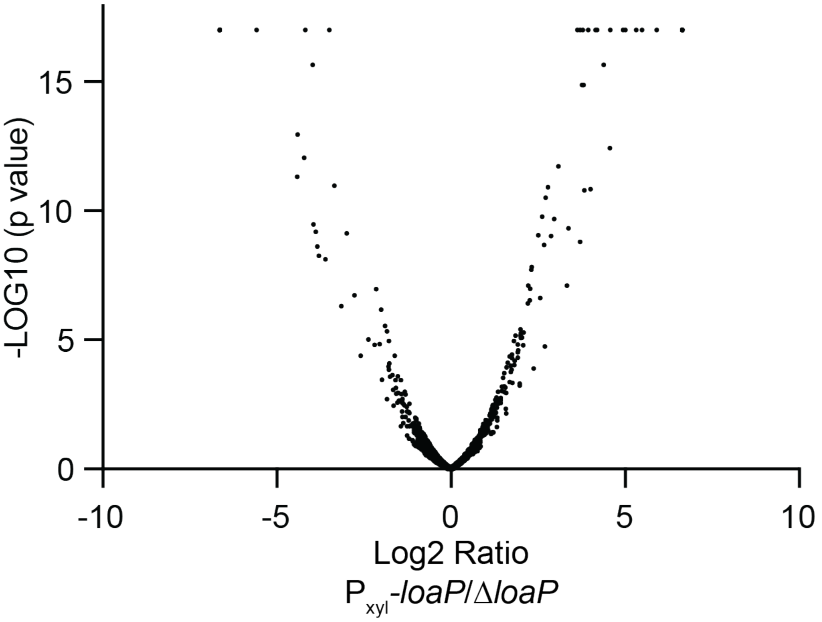

Supplementary Figure 1. Volcano plot comparing protein abundances between a LoaP overexpression strain (*loaP*+) and a Δ*loaP* background determined by mass spectrometry.

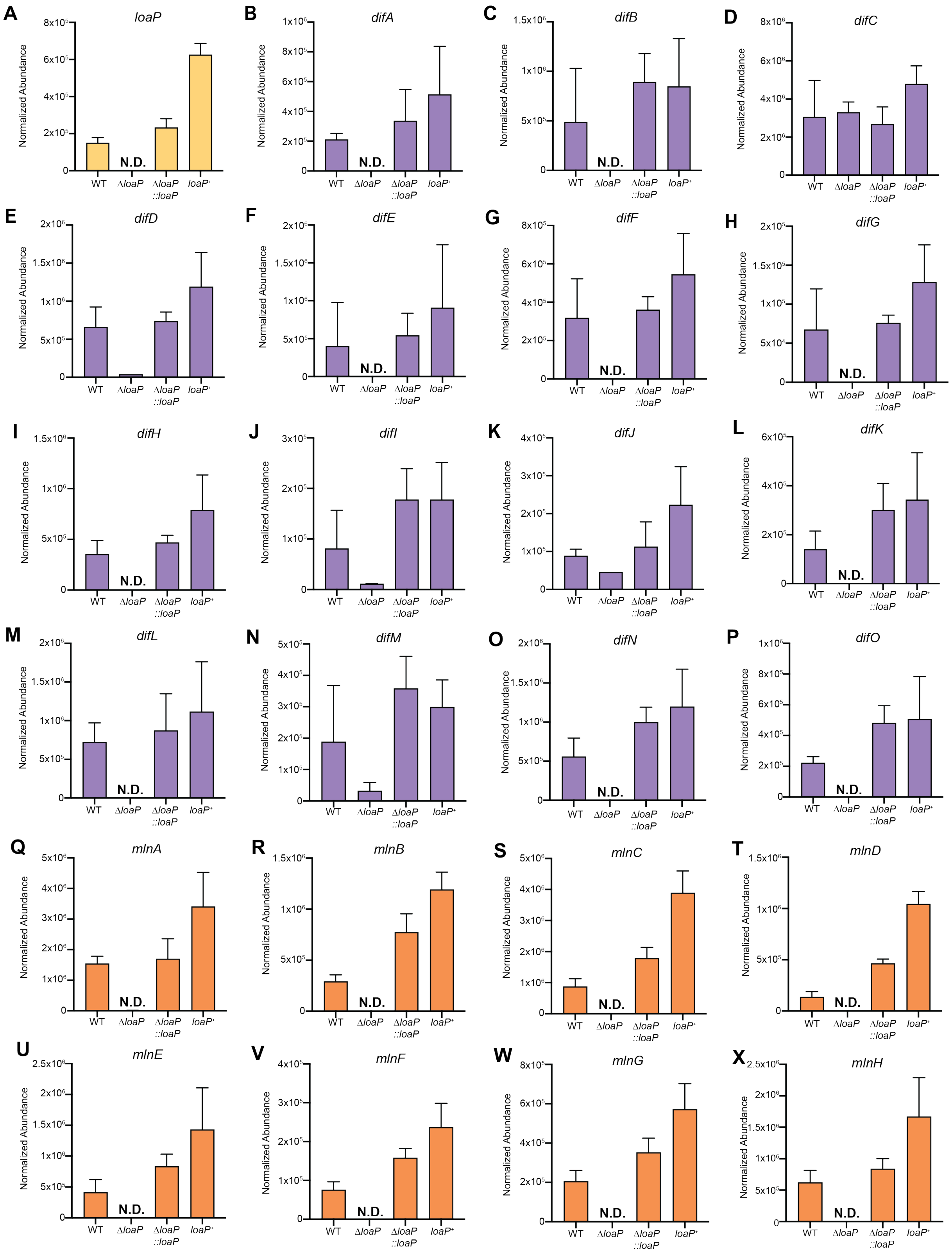

A loaP
B difA
C difB
D difC
E difD
F difE
G difF
H difG
I difH
J difI
K difJ
L difK
M difL
N difM
O difN
P difO
Q mlnA
R mlnB
S mlnC
T mlnD
U mlnE
V mlnF
W mlnG
X mlnH
Normalized Abundance
WT
ΔloaP
ΔloaP ::loaP
loaP⁺
N.D.

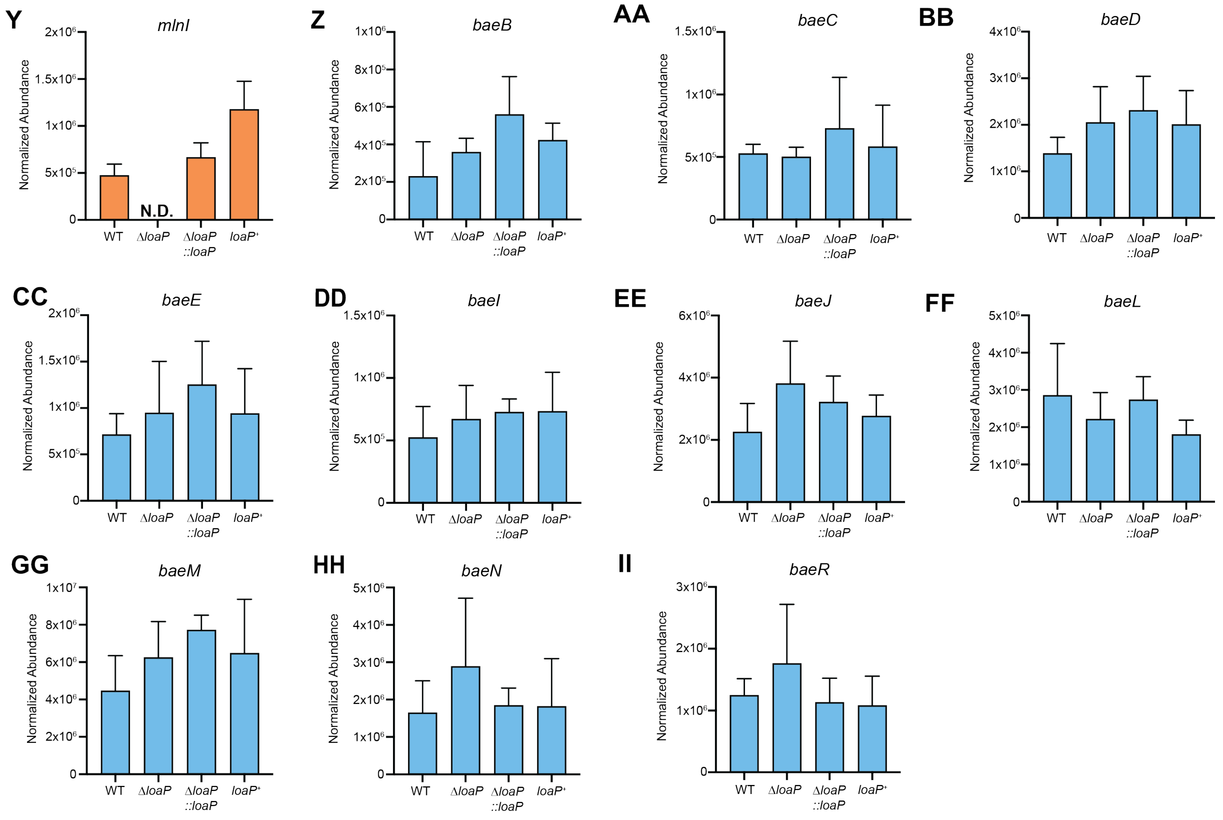

Supplementary Figure 2. Abundance of unique peptides for proteins encoded by PKS operons. **(A)** Abundance of unique peptides for LoaP. Abundances of unique peptides for proteins encoded by the *dfn* **(B-P)** *mln* **(Q-Y)** and *bae* **(Z-II)** operons, determined by mass spectrometry analysis of wild-type *B. velezensis* (WT), Δ*loaP*, a LoaP complementation strain (Δ*loaP::loaP*) or a LoaP overexpression strain (*loaP*$^{+}$) that contains two copies of the *loaP* gene. N.D, not detected.

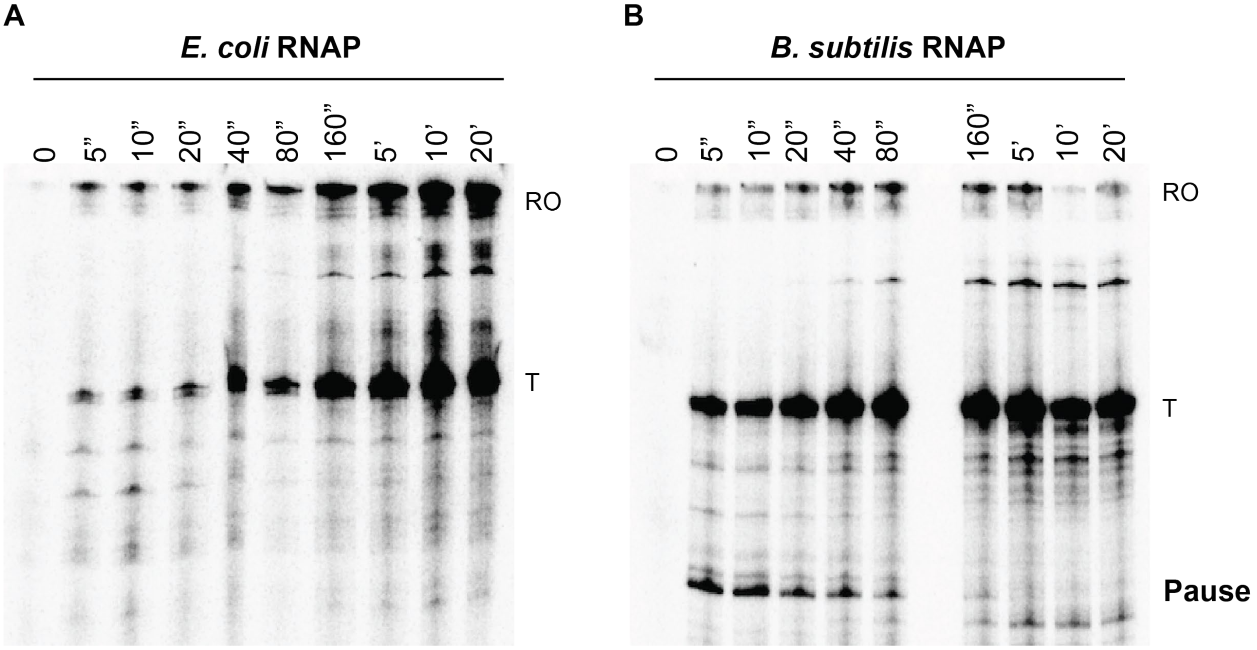

Supplementary Figure 3. A pause in the *dfn* leader region is specific to *Bs*RNAP **(A)** In vitro synchronized transcription of the *dfn* leader with *E. coli* RNAP **(B)** and *B. subtilis* RNAP. Aliquots were removed and quenched in loading buffer at the specified time points. Bands representing the pause site, termination (T) and run-off (RO) are specified.

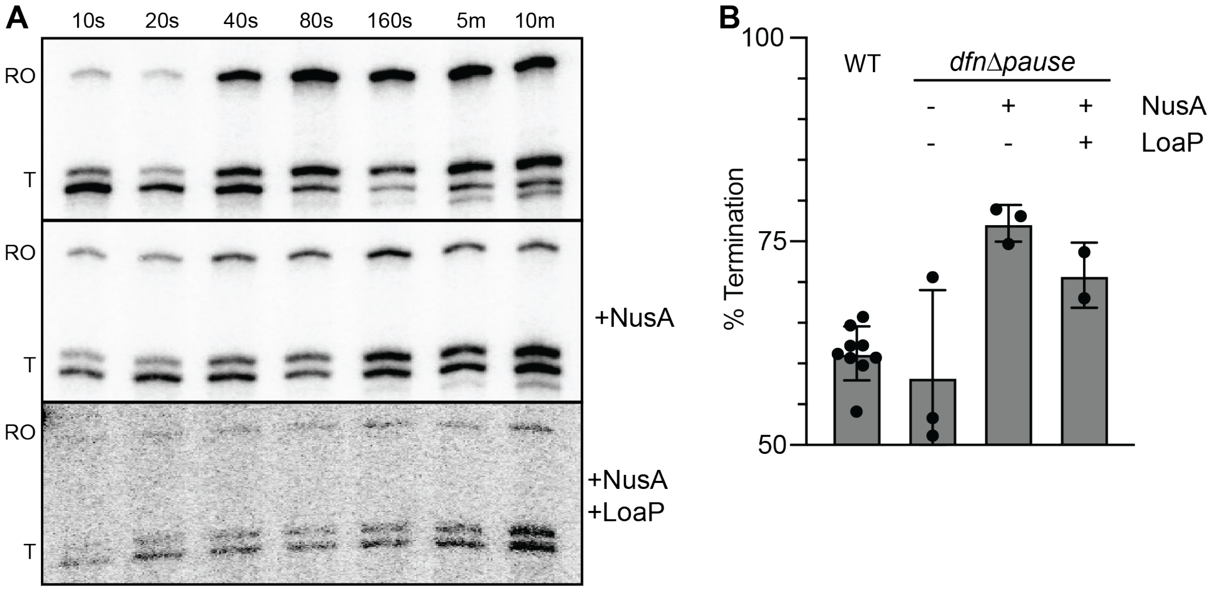

Supplementary Figure 4. Deletion of the *dfn* leader pause sequence in vitro **(A)** Time course in vitro transcription of Δ*pause* templates following the addition of NusA and/or LoaP (at 1 µM final), as specified. Reactions were incubated at 37 °C for 15 minutes prior to the addition of NTPs (200 µM), after which aliquots were removed at the specified time points and quenched in Stop buffer before being resolved by denaturing PAGE. **(B)** Quantification of transcription reactions shown in (a), measuring termination products as a normalized ratio of overall lane intensity and compared to a wild-type template.

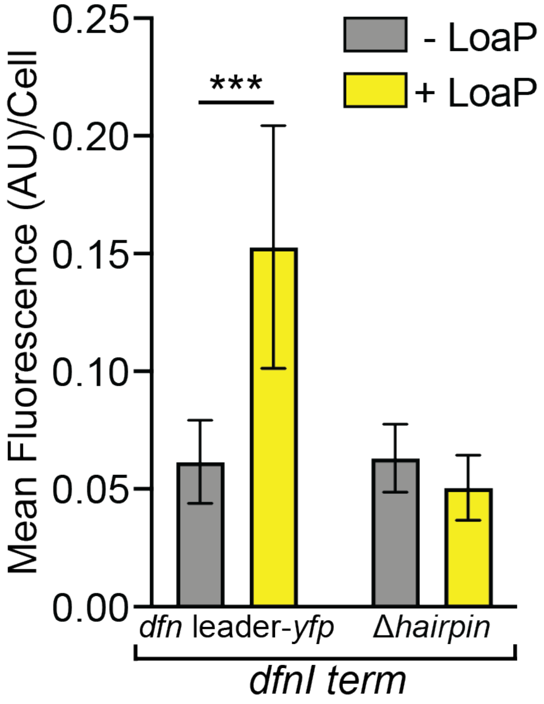

Supplementary Figure 5. LoaP antitermination at the *dfnI* terminator requires the *dfn* hairpin. Bar graph representing the average fluorescence intensity per cell measured from *B. subtilis* expressing a constitutive P_{const}-*dfn* leader-*yfp* reporter, or P_{const}-*dfn* leader Δhairpin -*yfp*, containing the *dfnI* terminator sequence directly upstream of *yfp*, as well as a xylose-inducible copy of *loaP* integrated into the nonessential *thrC* locus. Statistical significance was determined following unpaired t tests. Three asterisks (***) denotes a p-value < 0.001.

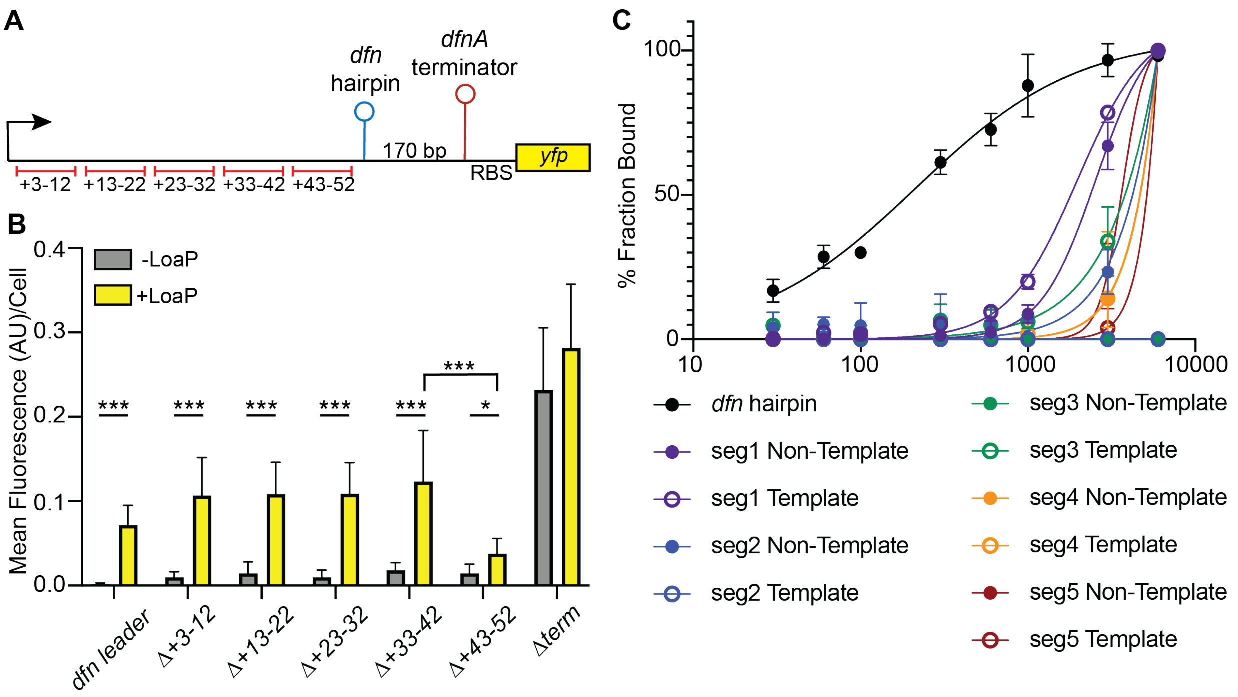

Supplementary Figure 6. Investigation of LoaP recruitment to the TEC **(A)** Schematic of the deletions made to the *dfn-yfp* reporter construct. The *dfn* hairpin is represented in blue and the *dfnA* terminator in red. Deletions of 10 bp DNA segments are denoted by red bars. **(B)** Bar graph representing the average fluorescence intensity per cell measured from *B. subtilis* expressing a constitutive P_{const}-*dfn* leader-*yfp* reporter possessing the deletions specified, as well as a xylose-inducible copy of *loaP* integrated into the nonessential *thrC* locus. **(C)** Equilibrium binding curves obtained via DRaCALA wherein increasing amounts of LoaP were combined with radiolabeled synthetic DNAs. The normalized fraction bound is shown with error bars representing the standard deviation from the mean. Binding affinities were calculated from at least three experimental replicates.

Supplemental Table 1. Strains used in this study

Strain Name	Strain Background	Genotype	Antibiotic Marker	Source
FZB42	*B. velezensis* FZB42	Wild-type *B. velezensis*		
JG091	*B. velezensis* FZB42	*loaP::erm*	Erm	Goodson, 2017
JG098	*B. velezensis* FZB42	*amyE*:: Pxyl-*loaP*	Cm	Goodson, 2017
JG099	*B. velezensis* FZB42	*loaP::erm*<br>*amyE*:: Pxyl-*loaP*	Erm, Cm	Goodson, 2017
MDJ174	*B. velezensis* FZB42	*loaP::erm*<br>*amyE*:: Pxyl-*loaP* E40A	Erm, Cm	*This study*
MDJ175	*B. velezensis* FZB42	*loaP::erm*<br>*amyE*:: Pxyl-*loaP* R41A	Erm, Cm	*This study*
MDJ189	*B. velezensis* FZB42	*loaP::erm*<br>*amyE*:: Pxyl-*loaP* K128A	Erm, Cm	*This study*
MDJ190	*B. velezensis* FZB42	*loaP::erm*<br>*amyE*:: Pxyl-*loaP* I143A	Erm, Cm	*This study*
JG257	*B. subtilis* 168	*thrC::*Pxyl-*loaP*<br>*amyE::*Pconst-*dfn-yfp*	Erm, Cm	Elghondakly, 2021
MDJ330	*B. subtilis* 168	*thrC::*Pxyl-*loaP*<br>*amyE::*Pconst-*dfnΔhp-yfp*	Erm, Cm	*This study*
MDJ331	*B. subtilis* 168	*thrC::*Pxyl-*loaP*<br>*amyE::*Pconst-*dfnΔpause-yfp*	Erm, Cm	*This study*
MDJ332	*B. subtilis* 168	*thrC::*Pxyl-*loaP*<br>*amyE::*Pconst-*dfnΔterm-yfp*	Erm, Cm	*This study*
MDJ334	*B. subtilis* 168	*thrC*::Pxyl-*loaP*, *amyE*::Pconst-*dfnΔ+3-12-yfp*	Erm, Cm	*This study*
MDJ335	*B. subtilis* 168	*thrC*::Pxyl-*loaP*, *amyE*::Pconst-*dfnΔ+13-22-yfp*	Erm, Cm	*This study*
MDJ336	*B. subtilis* 168	*thrC*::Pxyl-*loaP*, *amyE*::Pconst-*dfnΔ+23-32-yfp*	Erm, Cm	*This study*
MDJ337	*B. subtilis* 168	*thrC*::Pxyl-*loaP*, *amyE*::Pconst-*dfnΔ+33-42-yfp*	Erm, Cm	*This study*
MDJ338	*B. subtilis* 168	*thrC*::Pxyl-*loaP* *amyE*::Pconst-*dfnΔ+43-52-yfp*	Erm, Cm	*This study*
MDJ345	*B. subtilis* 168	*amyE*::Pconst-*dfn-yfp*<br>pMJ106(Pxyl-*nusA*)	Erm, Cm	*This study*
MDJ347	*B. subtilis* 168	*amyE::*Pconst-*dfnΔterm-yfp*<br>pMJ106(Pxyl-*nusA*)	Erm, Cm	*This study*

Supplemental Table 2 Synthetic nucleic acid species used in this study

Oligo	Sequence	Description
Amr261	GGAAAGGCAAUCGCUGCUUCGGCACGUUGCC	*dfn* hairpin with 5' GGAAA extension (unpaired)
Amr282	GGAAAGGGCCCUGAAGAAGGGCCC	*boxB* hairpin with 5' GGAAA extension (unpaired)
dfnA term	GGAAACACAAUGCGUUUGCGAUUUUUAAACCUGUGCUGAAUCGCAAACGCGUUUGUG	dfnA terminator with 5' GGAAA extension (unpaired)
dfnA mut	GGAAACACAGUUUGCGAUUUUUAAACCUGUGCUGAAUCGCAAACUGUG	dfnA terminator mutant with 5' GGAAA extension (unpaired)
MDJ652	AATAGCAACT	*dfn* segment 1 NT
MDJ653	ATTTGTTAAA	*dfn* segment 2 NT
MDJ654	TTTATTAAAT	*dfn* segment 3 NT
MDJ655	TGATGTAAAG	*dfn* segment 4 NT
MDJ656	CACCTATTAA	*dfn* segment 5 NT
MDJ657	AGTTGCTATT	*dfn* segment 1 T
MDJ658	TTTAACAAAT	*dfn* segment 2 T
MDJ659	ATTTAATAAA	*dfn* segment 3 T
MDJ660	CTTTACATCA	*dfn* segment 4 T
MDJ661	TTAATAGGTG	*dfn* segment 5 T

Other Sequences:

dfn leader transcription template:

AATACTGAATTGTAAGGATCCAAGAACAGC**TTGACAAATACACAAGAGTGTGTTATAATGCAATT**
AGAATGAGTTGAGTTAGAGAATAGGGTAGCGAAAGCTACCCTCGAGTTaaatagcaactatttgttaaatt
tattaaattgatgtaaagcacctattaaa**(tttcaatcgctgtttcggcacgttgaaga)**ggaaatagggattataaaatgatt
gtagtgtatatatctacatatacatgataagggagaagtgtg**[ccgtcatggctgttttcgggcatatt]**ggcactgcaacttgt
catgcgaaacattcagtaaacaagcaagatgtctttatataaattatgttagaaaaggaaaa*cacaatgcgtttgcgatttttaa*
*acctgtgctgaatcgcaaacgcgtttgtgtttttattttt*ATGCCGGTGTTTACACCAGTCGAATTCAAGAACGAAT
TCAAGCTTGGCACTGGCCGTCGTTTTACAACGTCGTGAC

Key:

Promoter

C-less cassette

Transcription start site

dfn leader region (through *terminator*)

(*dfn* hairpin)

[pause site]
